# An integrated comparative genomics, subtractive proteomics and immunoinformatics framework for the rational design of a Pan-*Salmonella* multi-epitope vaccine

**DOI:** 10.1371/journal.pone.0292413

**Published:** 2024-07-03

**Authors:** Arittra Bhattacharjee, Md. Rakib Hosen, Anika Bushra Lamisa, Ishtiaque Ahammad, Zeshan Mahmud Chowdhury, Tabassum Binte Jamal, Md. Mehadi Hasan Sohag, Mohammad Uzzal Hossain, Keshob Chandra Das, Chaman Ara Keya, Md Salimullah

**Affiliations:** 1 Bioinformatics Division, National Institute of Biotechnology, Ganakbari, Ashulia, Savar, Dhaka, Bangladesh; 2 Department of Genetic Engineering and Biotechnology, Jagannath University, Dhaka, Bangladesh; 3 Molecular Biotechnology Division, National Institute of Biotechnology, Ganakbari, Ashulia, Savar, Dhaka, Bangladesh; 4 Department of Biochemistry and Microbiology, North South University, Bashundhara, Dhaka, Bangladesh; The University of Texas Medical Branch at Galveston, UNITED STATES

## Abstract

Salmonella infections pose a significant global public health concern due to the substantial expenses associated with monitoring, preventing, and treating the infection. In this study, we explored the core proteome of *Salmonella* to design a multi-epitope vaccine through Subtractive Proteomics and immunoinformatics approaches. A total of 2395 core proteins were curated from 30 different isolates of *Salmonella* (strain NZ CP014051 was taken as reference). Utilizing the subtractive proteomics approach on the *Salmonella* core proteome, Curlin major subunit A (CsgA) was selected as the vaccine candidate. *csgA* is a conserved gene that is related to biofilm formation. Immunodominant B and T cell epitopes from CsgA were predicted using numerous immunoinformatics tools. T lymphocyte epitopes had adequate population coverage and their corresponding MHC alleles showed significant binding scores after peptide-protein based molecular docking. Afterward, a multi-epitope vaccine was constructed with peptide linkers and Human Beta Defensin-2 (as an adjuvant). The vaccine could be highly antigenic, non-toxic, non-allergic, and have suitable physicochemical properties. Additionally, Molecular Dynamics Simulation and Immune Simulation demonstrated that the vaccine can bind with Toll Like Receptor 4 and elicit a robust immune response. Using *in vitro*, *in vivo*, and clinical trials, our findings could yield a Pan-*Salmonella* vaccine that might provide protection against various *Salmonella* species.

## 1. Introduction

*Salmonella* species are a type of Gram-negative, rod-shaped, facultative anaerobic bacteria belonging to the Enterobacteriaceae family [1]. The global prevalence of *Salmonella* infection is a significant public health issue that contributes to the economic burden experienced by both developed and developing nations. This cost arises from the expenses associated with illness surveillance, prevention, and treatment [2]. The majority of *Salmonella* infections in individuals derive from the ingestion of food or water that has been contaminated with the pathogen. *Salmonella* is the most common source of foodborne illness globally, with an estimated 80.3 million cases annually. The most common form of salmonellosis ranges from gastroenteritis (diarrhea, abdominal cramps, and fever) to life-threatening enteric fevers [3].

There are two known species of *Salmonella*: *Salmonella bongori* and *Salmonella enterica*. The *S*. *enterica* can be further divided into six subspecies, specially referred to as enterica (serotype I), salamae (serotype II), arizonae (IIIa), diarizonae (IIIb), houtenae (IV), and indica (VI). *S*. *bongori* and *S*. *enterica* consist of over 2600 serovars [4]. Based on their clinical manifestations, *Salmonella* strains can be categorized into typhoid *Salmonella* and non-typhoid *Salmonella* (NTS). Typhoid *Salmonella* mainly comprises *Salmonella enterica* serovars, *S*. Typhi and *S*. Paratyphi which are responsible for causing fever, collectively referred to as enteric fever (typhoid and paratyphoid fever) [5]. Humans are the natural reservoir of *Salmonella* Typhi and *Paratyphi A*. Typhoid and paratyphoid infections primarily infect blood, with typical symptoms including headache, malaise, prolonged high fever, gastrointestinal bleeding, altered mental states (the typhoid state), ileus, septic shock, intestinal perforation, and death [6]. Children are the major sufferers of typhoid and paratyphoid infections, particularly in South Asia, Southeast Asia, and Sub-Saharan Africa, due to poor access to clean water and sanitation. Enteric fever results in 200,000 fatalities and 11–21 million illnesses worldwide each year, with the majority of cases occurring in low-income countries [7].

*Salmonella* infections are also a major cause of gastroenteritis, a disease accounting for 93.8 million cases worldwide every year with 155,000 deaths [8]. Gastroenteritis is an infection of the colon and terminal ileum that produces diarrhea, vomiting, and abdominal cramps [9]. *Salmonella* infection is also posing a serious threat to the poultry industry and other livestock industries, as the strains can be found in both domestic and wild animals, including dogs, cats, reptiles, and rodents. Close contact with *Salmonella*-infected humans or animals can lead to infection. Hence, reservoir diversity is a major threat to controlling the infections [10,11].

Antibiotics are currently used for the treatment of severe *Salmonella* infections. However, the emergence and re-emergence of multidrug-resistant *Salmonella* raises a serious obstacle to handling *Salmonella* infections [12]. A variety of processes, including horizontal transfer of resistance genes, and inappropriate use of antibiotics in human and veterinary medicine, as well as in agriculture, have contributed to the development and spread of resistance in *Salmonella* strains [13]. Multidrug-resistant *Salmonella* infections can have serious consequences, as treatment choices become limited or ineffective. Infections can cause prolonged sickness, hospitalization, and even death in some circumstances, especially in susceptible groups such as the elderly, small children, and people with weaker immune systems [14]. The development of vaccines that will be effective against these resistant strains as well as against a broad spectrum of *Salmonella* infections is imperative. Undoubtedly, immunoinformatics can greatly aid in this quest.

Immunoinformatics is an interdisciplinary domain that integrates the fields of immunology and bioinformatics, utilizing computational methods to analyze immune-related data and aid in the design of vaccines, therapeutics, and diagnostics [15]. Immunoinformatics approaches to vaccine design offer several advantages, including speed, efficiency, and targeted selection of vaccine candidates. By utilizing computational methods, researchers can predict immunogenicity, identify epitopes, optimize vaccine design, assess cross-reactivity, and prioritize vaccine targets. This accelerates the vaccine development process, improves safety, and enhances the likelihood of developing effective immunization strategies [16]. The immunoinformatics approach has been used to develop vaccines against a variety of infectious agents, including, SARS-CoV 2, the Human Immunodeficiency Virus (HIV-1), the Ebola virus, the *Herpes Simplex* Virus (HSV)-1 and 2, the human norovirus, the *Venezuelan equine encephalitis* virus, the Sudan virus, *Staphylococcus aureus*, *Shigella spp*., and others [17,18].

The objective of the study was to design a multi-epitope vaccine against broad-spectrum *Salmonella* spp. infections by exploring the core proteome of *Salmonella* and adapting subtractive proteomics and immunoinformatics approaches.

## 2. Methods

### 2.1. Retrieval of the core proteome and removal of paralogous proteins

The core proteomes of *Salmonella* spp. were retrieved from the Efficient Database framework for comparative Genome Analyses using BLAST score Ratios (EDGAR) version 3.0 [19] (S1 Table). The 16s rRNA genes of all 30 strains were aligned using Multiple Alignment using Fast Fourier Transform (MAFFT) (https://mafft.cbrc.jp/). *Shigella flexneri* strain ATCC 29903 (NCBI accession number: NR_026331.1) 16s rRNA gene was taken as an outgroup. The alignment file was used to construct a phylogenetic tree using the Neighbor-Joining Method with bootstrap (value: 1000) [20]. The tree was visualized by iTOL [21]. A circular plot of three distantly related strains was selected to demonstrate a portion of the core genome. The plot was generated by BioCircos [22]. The core proteome was collected from the core genome dataset. To ensure the removal of paralogous protein sequences from that core proteome, the Cluster Database at High Identity with Tolerance (CD-HIT) tool was applied. A threshold value of 0.6 (60%) was applied to determine all the paralogous sequences [23].

### 2.2. Removal of host homologous proteins and identification of essential pathogenic proteins

To identify the proteins that are non-homologous to the host (*Homo sapiens*), a comparative protein Basic Local Alignment Search Tool (BLASTp) analysis of the selected non-paralogous proteins was performed against the National Center for Biotechnology Information (NCBI) Human Proteome (NCBI: taxid9606). The expectation value (E-value) cut-off was set to 10^−4^ and the sequence similarity cut-off was set to 50%. Following the assessment of various parameters, proteins exhibiting significant similarity were excluded from further consideration, leaving only those that were non-homologous for further investigation. The Database of Essential Genes (DEG) serves as a repository containing genes essential for all organisms [24]. The non-homologous proteins were employed in BLASTp against the data of DEG to identify essential genes that are vital for the survival of the bacteria. The E-value threshold for the analysis was <0.0001 [25].

### 2.3. Identification of unique metabolic pathways

The Kyoto Encyclopedia of Genes and Genomes (KEGG) database was used to identify the unique metabolic pathways present only in the pathogen. KEGG Automatic Annotation Server (KAAS) performs BLASTp similarity searches of all non-homologous essential proteins against a periodically updated KEGG database to identify unique metabolic pathways [26].

### 2.4. Prediction of subcellular localization and selection of vaccine candidate

The subcellular location of a protein is important for identifying appropriate vaccine targets [27]. To predict subcellular localizations of the proteins PSORTb version 3.0 was used (https://www.psort.org/psortb/) [28]. The extracellular and outer membrane proteins, derived through subcellular localization, were subjected to NCBI BLASTp against the human gut microbiota (NCBI taxid:408170). Based on the BLAST results, one extracellular protein was selected as a vaccine candidate.

### 2.5. Antigenicity, conservancy, and transmembrane topology prediction

The antigenicity of the protein was determined using ANTIGENpro (https://scratch.proteomics.ics.uci.edu/) and VaxiJen (http://www.ddgpharmfac.net/vaxijen/VaxiJen/VaxiJen.html). Unipro UGENE software (http://ugene.net/) was used to show the conservancy of the CsgA protein sequences within 30 different strains of *Salmonella* [29]. The TMHMM server utilizes a hidden Markov model to accurately predict transmembrane helices. The transmembrane helices residues and outside amino acids of the vaccine candidate were predicted using the TMHMM 2.0 server [30].

### 2.6. Prediction of B cell and T cell epitopes

The identified outer sequence of the vaccine candidate was then submitted to the BepiPred 2.0 server (https://services.healthtech.dtu.dk/services/BepiPred-2.0/) for the prediction of B cell epitopes [31]. Prioritizing surface-exposed amino acids, most potential B cell epitopes were selected [32]. The Cytotoxic T cell (CTL) epitopes for 12 classes of Major Histocompatibility Complex 1 (MHC 1) supertype were identified using NetCTL 1.2 (https://services.healthtech.dtu.dk/services/NetCTL-1.2/) [33]. The threshold for these 9-mer CTL epitope predictions was 0.75 and the MHC 1 supertypes were A1, A2, A3, A24, A26, B7, B8, B27, B39, B44, B58, and B62. Based on the combined score, the most effective CTL epitopes were chosen. The 15-mer Helper T cell (HTL) epitopes for Human Leukocyte Antigen (HLA)-DP, HLA-DQ, and HLA-DR alleles were predicted using NetMHCII (https://services.healthtech.dtu.dk/services/NetMHCII-2.3/). Considering the affinity score, percentage ranking, and binding strength most potential HTL epitopes were selected [34].

### 2.7. Peptide modeling and molecular docking between T lymphocyte epitopes and MHC alleles

Epitopes that exhibit robust interaction with their respective MHC alleles are considered suitable candidates for the development of multi-epitope vaccines. A total of 5 CTL epitopes and 2 HTL epitopes were selected for docking with MHC alleles based on their combined score and binding strength. At first, the corresponding MHC alleles of these epitopes were retrieved from the Research Collaboratory for Structural Bioinformatics (RCSB) Protein Data Bank (PDB) (https://www.rcsb.org/) and processed using BIOVIA Discovery Studio to remove unnecessary ligands (https://discover.3ds.com/) The respective PDB IDs of the selected epitopes was HLA A1 (3BO8), HLA A24 (5XOV), HLA B39 (4O2E), HLA B58 (5VWJ), HLA DQ (7KEI), HLA DR (1AQD), and UniProtKB id for HLA A26 is Q5SPM2 (only predicted structure available). The PEP-FOLD 3.5 server was employed to generate the three-dimensional (3D) structures of the selected CTL and HTL epitopes [35]. The primary objective of this server is to forecast the structural arrangement of short peptides composed of 5 to 50 amino acids. To achieve this, the server utilizes the Forward Backtrack/Taboo Sampling approach [57]. The docking interactions between epitopes and MHC molecules were implemented using Galaxy Tong Dock A and visualized by BIOVIA Discovery Studio.

### 2.8. Population coverage analysis

Population coverage analysis helps to determine the percentage of people in a specific area who will have an immune response to the vaccine. The geographical distribution of a vaccine’s efficacy is dependent upon the diversity of the MHC alleles that its epitopes are capable of recognizing. Hence, the IEDB population coverage analysis tool was employed to estimate the global collective coverage of the chosen HTL and CTL epitopes [36].

### 2.9. Construction of multi-epitope vaccine

The selected B cell and T cell epitopes were connected by GPGPG and AAY linkers to construct the vaccine sequence [37]. Human Beta Defensin-2 (HBD-2) (PDB ID: 1FD3) was conjugated at the end of the sequence because of its ability to activate Toll-Like Receptor 4 (TLR 4) [38]. The B cell epitopes were joined by GPGPG linkers, while the B cell-T cell and T cell-T cell epitopes were linked by AAY linkers and finally, the EAAAK linker was employed to conjugate HBD-2. The linkers were chosen based on their length, rigidity, flexibility, and effectiveness, as demonstrated by previous studies [39].

### 2.10. Predicting antigenicity, toxicity, and allergenicity of vaccine construct

To induce an immune response and promote the formation of memory cells, it is imperative that the vaccine construct possess a high degree of antigenicity. Hence, the antigenicity of the 3D vaccine design was evaluated through the utilization of ANTIGENpro and VaxiJen 2.0 server [46,47]. The AllerTOP 2.0 and ToxinPred servers were employed to predict the allergenicity and toxicity of the vaccine design [40,41].

### 2.11. Physicochemical properties evaluation and tertiary structure prediction of vaccine

The physicochemical properties of the vaccine construct were evaluated using the ProtParam server [60]. These properties included the amino acid composition, molecular weight, theoretical isoelectric point (pI), aliphatic index (AI), in vitro and in vivo half-life, instability index (II), and grand average of hydropathicity (GRAVY) [42]. The tertiary structure of the vaccine was generated through the Alpha fold 2.0 server using ColabFold [43]. Refinement of the structure was performed via Galaxy refinement and 3D refine server [44,45]. To validate the structure Procheck, ProSAWeb, and ERRAT 3.0 were used [46–48].

### 2.12. Molecular docking of the vaccine with TLR4 receptor

The tertiary structure of TLR4 was retrieved from the RCSB PDB (PDB ID: 3FXI). The structure was processed with BIOVIA Discovery Studio to remove all the heterogeneous regions except chain A. Molecular docking between TLR4 and the vaccine was carried out using Galaxy Tong Dock A. The most suitable model was selected by considering both the score and cluster size.

### 2.13. Immune simulation study

The immune response profile of the vaccine was predicted by using the C-ImmSim server (https://prosa.services.came.sbg.ac.at/prosa.php) for *in silico* immune simulation. C-ImmSim uses a position-specific scoring matrix (PSSM) and machine learning techniques for the prediction of immune responses [49]. The minimum time interval between two consecutive doses is 4 weeks [50]. Therefore, a total of three injections, each containing 1000 units of the vaccine, were administered at intervals of four weeks. The injections were given at time steps of 1, 84, and 168, respectively, with each time step being an equivalent of 8 hours in real life. In this simulation, the time-step was configured to 1050, while the remaining parameters were maintained at their default values.

### 2.14. Molecular dynamics simulation

After molecular docking, a 100 ns Molecular Dynamics (MD) simulation was executed for both apo-TLR4 and the vaccine-TLR4 complex using the GROningen MAchine for Chemical Simulations (GROMACS) (version 2020.6) (https://www.gromacs.org/) [51]. The proteins were surrounded by the Transferable Intermolecular Potential 3P (TIP3) water model [52]. The protein-water system was energetically minimized with the CHARMM36m force-field [53]. K^+^ and Cl^-^ ions were added to make the system neutral. Following the energy minimization, isothermal-isochoric (NVT) and isobaric (NPT) equilibrations were executed. Then, a 100 ns production MD simulation was done. To analyze the dynamic properties of the proteins, Root Mean Square Deviation (RMSD), root mean square fluctuation (RMSF), radius of gyration (Rg), and solvent accessible surface area (SASA) were measured.

### 2.15. Codon adaptation and visualization of cloning

JCat (Java Codon Adaptation Tool) was used to reversely translate the vaccine sequence and optimize the codon for the *E*. *coli* K12 strain [54]. Three more factors were chosen to make sure that the optimised DNA sequence did not have any rho-independent transcription termination, prokaryotic ribosome binding sites, or restriction enzyme cleavage sites‥ The sequence was modified by introducing XhoI and NdeI restriction sites at the N- and C-terminal ends, respectively. Additionally, the stop codons were added to the 3′ OH, or C-terminal end. Finally, the adapted nucleotide sequence was cloned into the *E*. *coli* pET28a (+) Plasmid Vector via SnapGene 6.2 tools (www.snapgene.com).

## 3. Results

**Fig 1:**
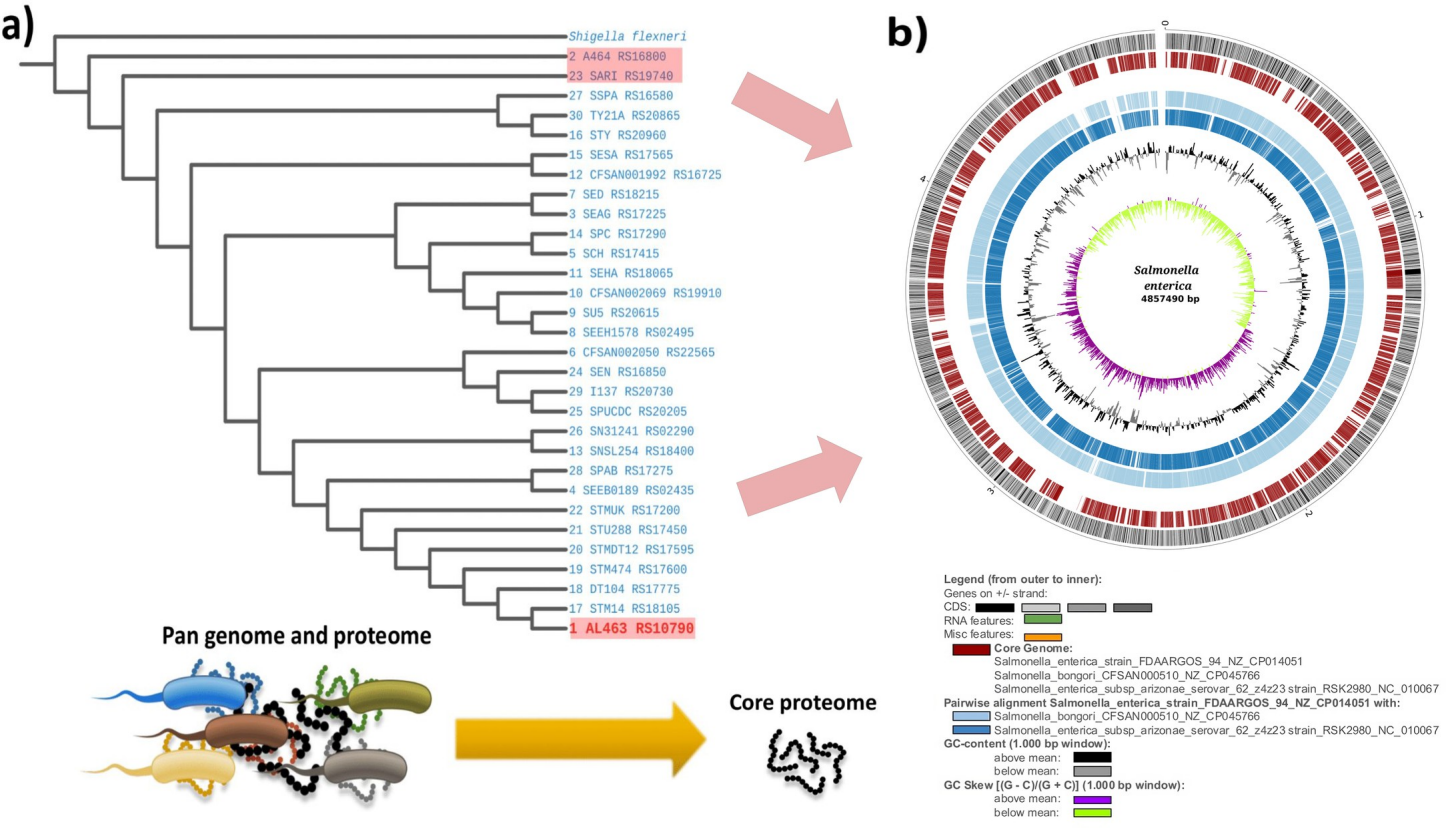
Complete workflow of the study.

An overview of the complete study is depicted in **Fig 1**.

### 3.1. Comparative genomics identified the core proteome of all the strains

**Fig 2:**
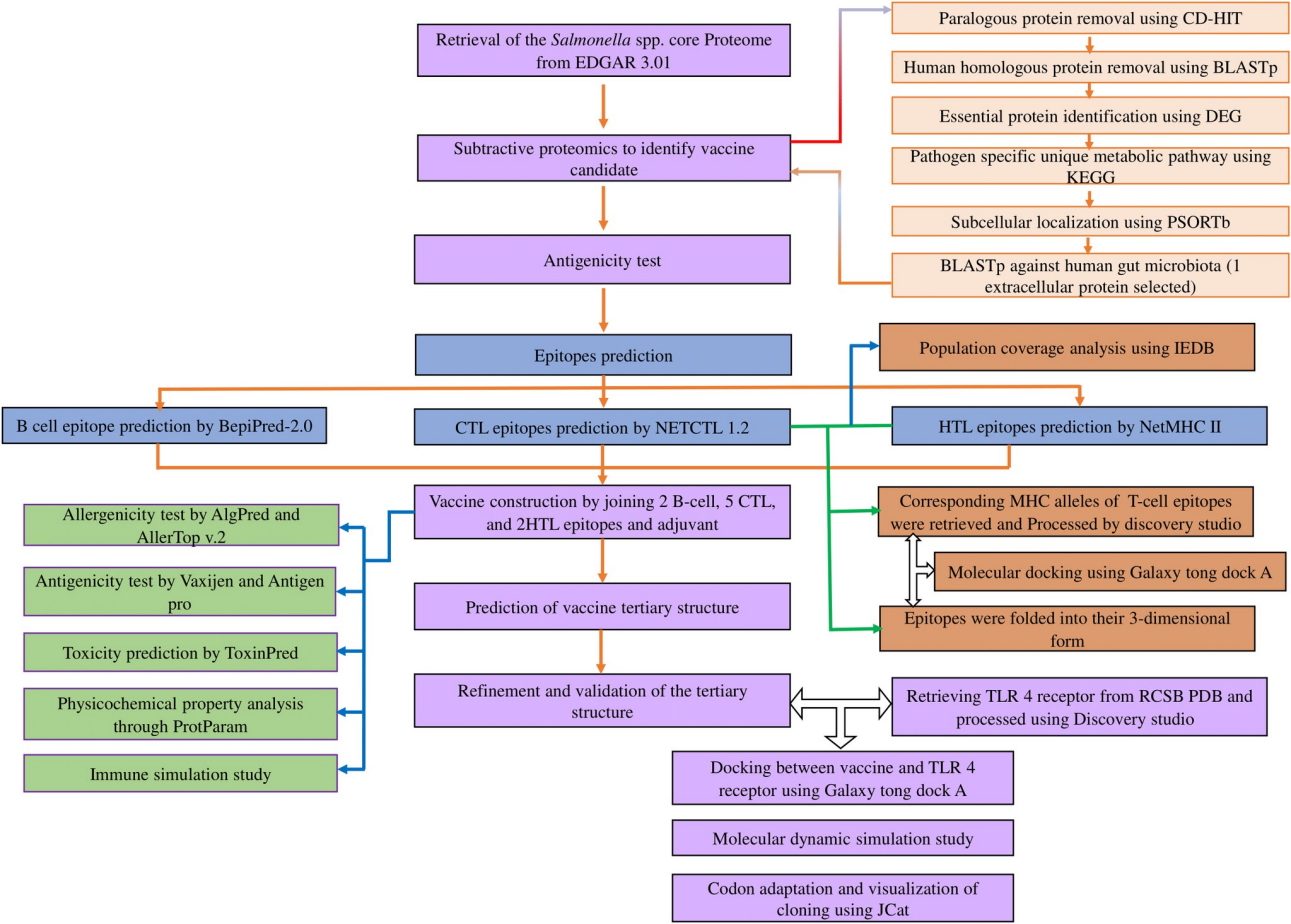
Thirty *Salmonella* strains were selected for the study. Here, a) a 16s rRNA-based phylogenetic tree of all the isolates (*Shigella flexneri* was taken as an outgroup), and b) a circular plot of three distantly related *Salmonella* species have been depicted. *Salmonella enterica* strain FDAARGOS_94_NZ_CP014051 was taken as a reference for the analysis.

The core genome distribution of the reference *Salmonella enterica* strain FDAARGOS_94_NZ_CP014051 is depicted **in Fig 2**. As the core proteome is present in most of the strains, vaccine constructs containing the core proteome formulation provide immune protection against a wide range of pathogenic species. A total of 2395 core protein sequences of 30 *Salmonella* strains were retrieved from EDGAR 3.0 (**S2 Table**).

### 3.2. Curlin major subunit (CsgA) is a suitable vaccine candidate

The core proteomes were subjected to CD-HIT to remove the paralogous sequences. The number of remaining non-paralogous sequences was 2386. The non-paralogous sequences were subjected to BLASTp to remove homologous sequences, leaving 1618 non-homologous sequences. The remaining proteins were screened for essential proteins and unique metabolic pathways, leaving only 793 sequences. These proteins were further analyzed for subcellular localization. PSORTb predicted 398 proteins as cytoplasmic, 35 as periplasmic, 231 as cytoplasmic membrane, 4 as extracellular, 20 as outer membrane, and the remaining 105 proteins’ locations were unknown (**S3 Table**). The 4 extracellular proteins were subjected to BLAST against the human gut microbiota. Among the proteins, the Curlin major subunit (CsgA) did not show any similarity against human gut microbiota proteome and hence was selected for further study as a vaccine candidate.

### 3.3. CsgA is antigenic and conserved among *Salmonella* strains

The selected CsgA protein showed an antigenic score of 0.941160 at ANTIGENpro and 1.0694 at VaxiJen, which suggested that the protein is highly antigenic which is evident by some previous studies [55]. Unipro UGENE showed the protein to be highly conserved among the 30 selected strains (**S1 Fig**). The transmembrane topology prediction tool TMHMM predicted that no transmembrane helices were present in the selected protein.

### 3.4. CsgA contains potential B and T cell epitopes

TMHMM predicted 126 outside amino acids in the selected protein. The outside residues were submitted to the BepiPred 2.0 server to predict B cell epitopes, which predicted four potential B cell epitopes (**S4 Table**). The NetCTL 1.2 server predicted a total of 29 unique CTL epitopes for 12 MHC class I supertypes that passed the threshold value of 0.75 for epitope identification (**S4 Table)**. Based on the combined score including proteasomal cleavage, TAP transport, and MHC-I binding efficiencies, five CTL epitopes (**Table 1**) were selected for vaccine construction. NetMHC II predicted HTL epitopes for HLA-DQ, DR, and DP. Considering the affinity score, percentage ranking, and binding strength, the two most potential HTL epitopes were selected (**Table 1**).

**Table 1:** List of selected epitopes for vaccine design.

B Cell epitopes	Epitopes sequence	Position
	GGGNHNGGGNSSGPD	2–16
	DQWNAKNSD	78–86
CTL epitopes	**Epitope Sequence**	**Combined Score**
	NSDITVGQY	3.4692
	QYGSANAAL	1.0441
	ETTITQSGY	2.2572
	QYGGNNAAL	1.3689
	RNNATIDQW	1.3572
HTL epitopes	**Epitope Sequence**	**Position**
	LSIYQYGSANAALAL	93–107
	SIYQYGSANAALALQ	94–108

### 3.5. The T cell epitopes showed decent binding capability with their corresponding MHC alleles

**Fig 3:**
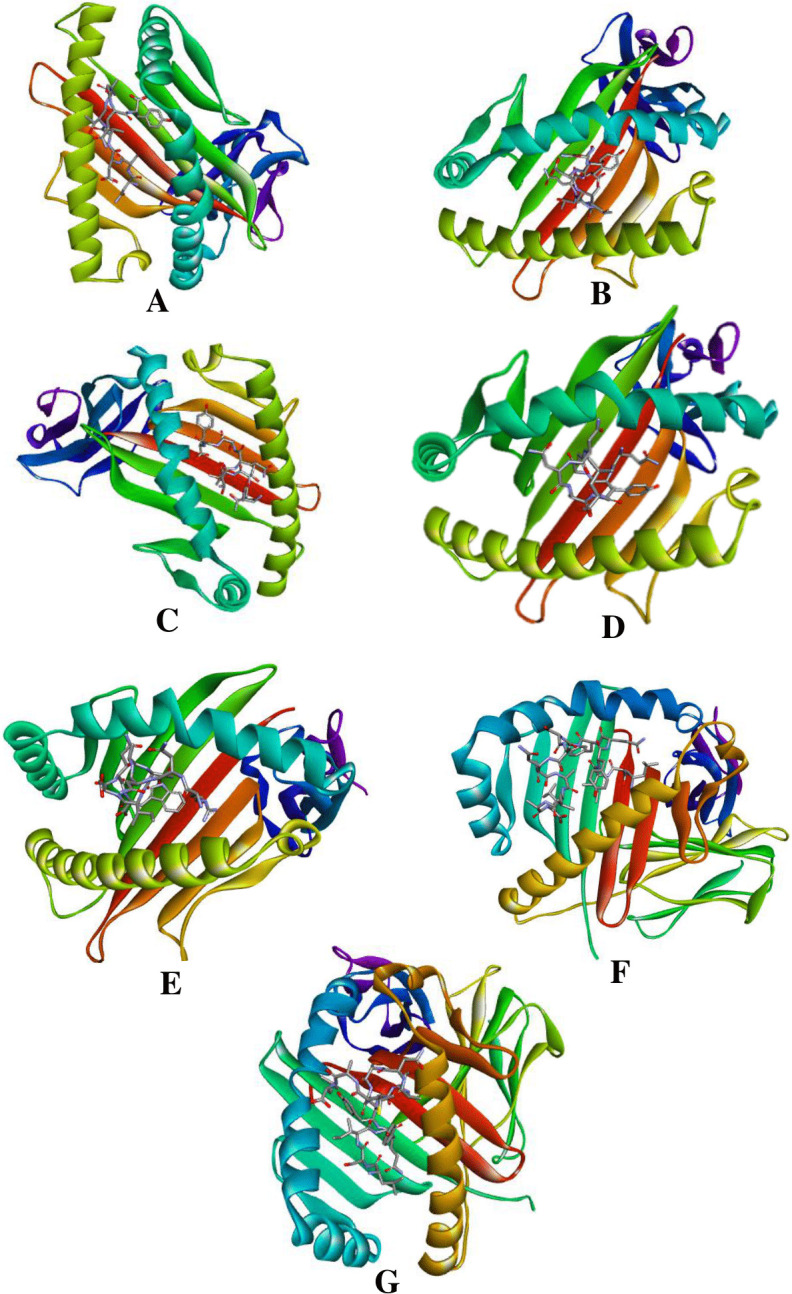
Molecular docking of HLA and corresponding epitopes. (A) HLA-A1- NSDITVGQY docking complex, (B) HLA-A24- QYGSANAAL, (C) HLA-A26- ETTITQSGY, (D) HLA-B39- QYGGNNAAL, (E) HLA-B58- RNNATIDQW, (F) DRB1-0101- LSIYQYGSANAALAL, (G) HLA-DQ- SIYQYGSANAALALQ.

Selected 7 T lymphocyte epitopes were docked against their corresponding MHC alleles. The docking result showed **(Table 2**) that all the epitopes are well interacting with their MHC alleles. The docking interactions are shown in **Fig 3**. Among the results, the best interaction was shown by CTL epitopes ETTITQSGY and MHC alleles HLA-A26 (**Fig 3C**). The docking score for this interaction was 910.597, and the cluster size was 393.

**Table 2:** Molecular docking of epitopes with corresponding MHC locus.

Docking complex	Docking Score	Cluster size
HLA-A1- NSDITVGQY	782.859	120
HLA-A24- QYGSANAAL	908.57	158
HLA-A26- ETTITQSGY	910.597	393
HLA-B39- QYGGNNAAL	816.048	231
HLA-B58- RNNATIDQW	812.447	319
DRB1-0101- LSIYQYGSANAALAL	882.502	99
HLA-DQ- SIYQYGSANAALALQ	967.499	114

### 3.6. The T cell epitopes should cover an adequate portion of the global population

**Fig 4:**
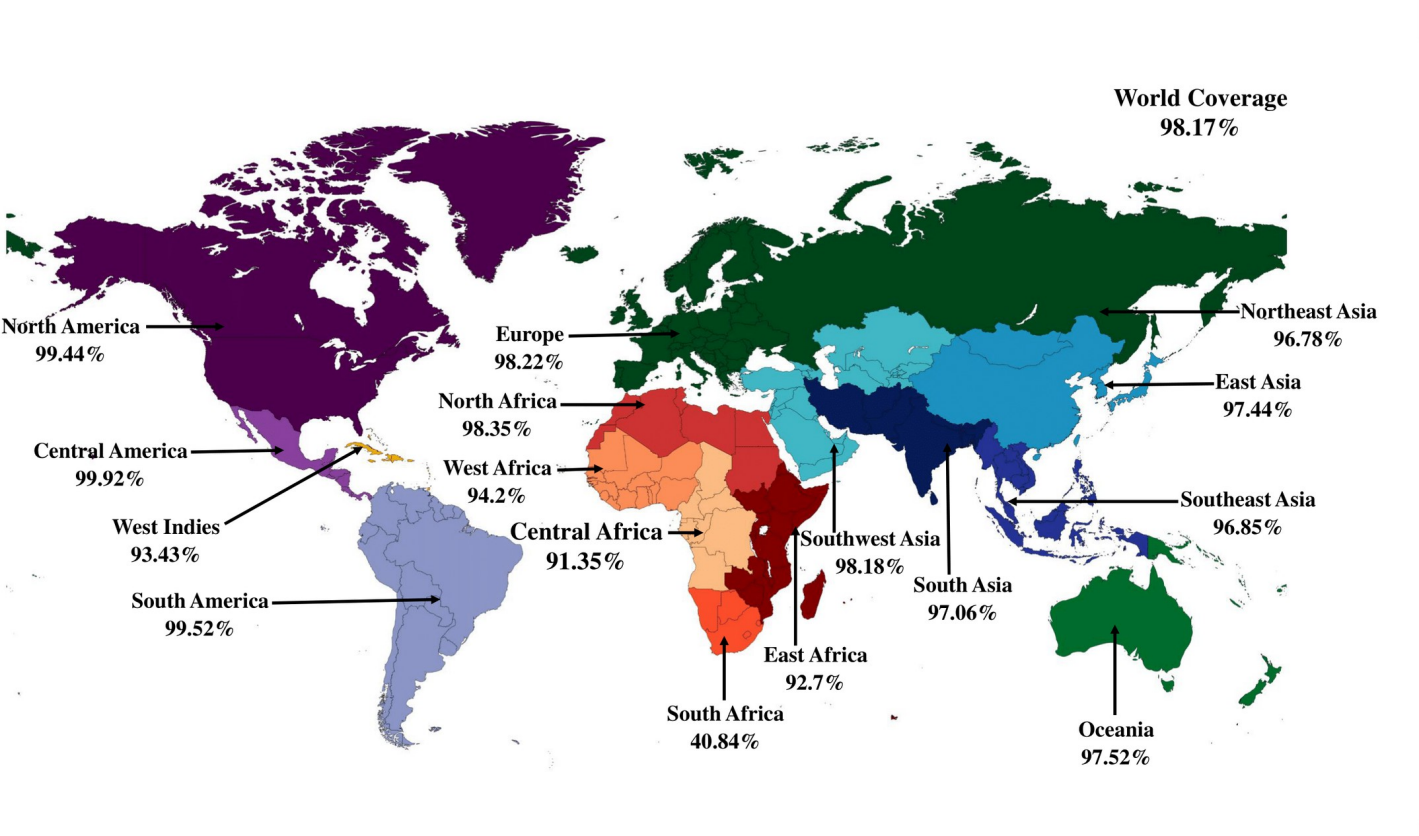
Worldwide population coverage of the selected T lymphocyte epitopes. Color variations represent different regions. Reprinted from [www.mapchart.net] under a CC BY license, with permission from the founder of MapChart, Minas Giannekas, original copyright *2023.

The predicted combined worldwide population coverage of our vaccine was 98.17%, and the average hit was 2.74 (**Fig 4**). Predictions showed that more than 98 percent of the population would respond to our vaccine.

### 3.7. The constructed multi-epitope vaccine was antigenic and non-allergenic

**Fig 5:**
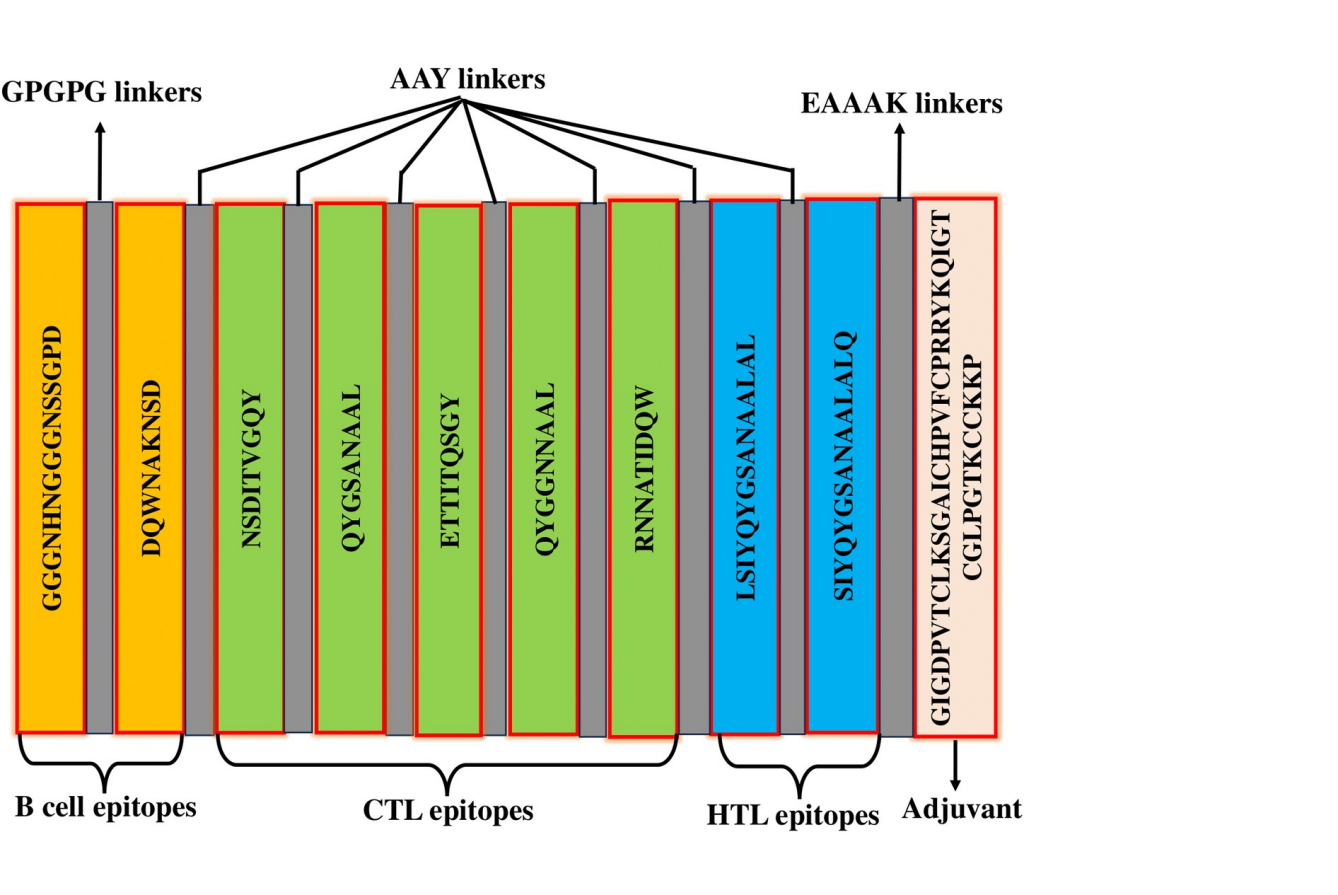
Schematic representation of the multi-epitope vaccine construct.

Our multi-epitope vaccine was constructed by combining two B-cell epitopes and seven T-cell (5 CTL and 2 HTL) epitopes joined by GPGPG and AAY linkers. HBD-2 was conjugated at the end of the sequence by the EAAAK linker. **Fig 5** illustrates the configuration of different epitopes and their corresponding linkers. The final vaccine construct comprises a total of 171 amino acid residues. The vaccine design was predicted to be highly antigenic by the ANTIGENpro and VaxiJen 2.0 servers, with scores of 0.871809 and 0.8028, respectively. According to the AllerTOP 2.0 prediction model, the vaccination was classified as non-allergenic. ToxinPred predicted 162 peptides in the vaccine in different combinations. Among these peptides, only six have toxic properties (**S5 Table**).

### 3.8. Physicochemical property evaluation and tertiary structure prediction

**Fig 6:**
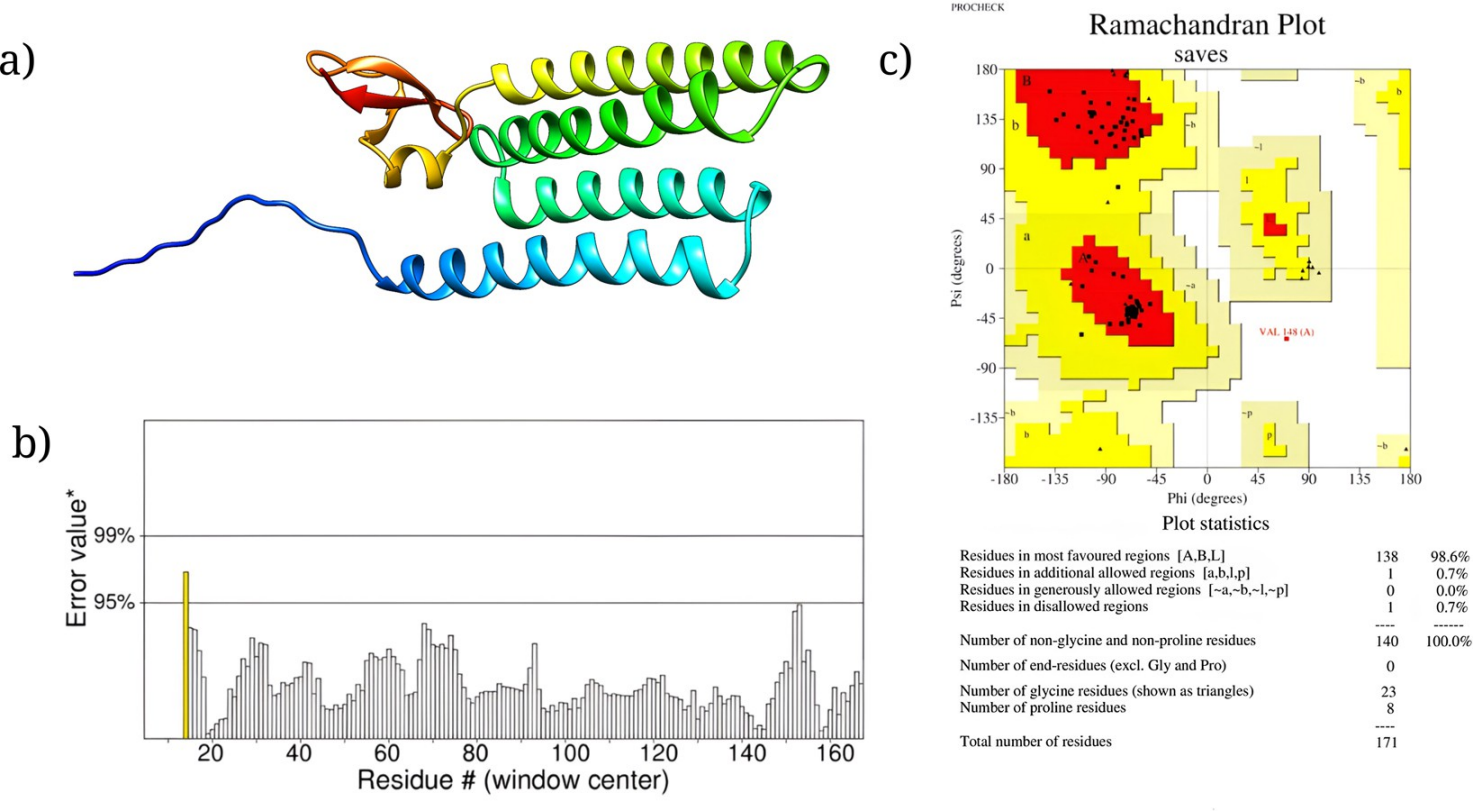
Validation of vaccine tertiary structure where (A) Vaccine tertiary structure, (B) Overall quality factor of the structure predicted by ERRAT and (C) Ramachandran plot are demonstrated.

The physicochemical characteristics of the vaccine construct are shown in **Table 3.** The vaccine had a molecular weight of 17543.29 Da, but its theoretical isoelectric point (pI) was determined to be 8.20. The computed instability index (II) showed a value of 22.44, suggesting that the protein under investigation exhibits stability. The thermostability of the vaccine was indicated by its aliphatic index (AI) value of 63.16. The estimated half-life for human reticulocytes *in vitro* was determined to be 30 hours, whereas for yeast *in vivo*, it was found to be greater than 20 hours, and for Escherichia coli *in vivo*, it was greater than 10 hours. The calculated Grand Average of Hydropathicity (GRAVY) value was determined to be -0.320, suggesting that the vaccine design had hydrophilic properties. The tertiary structure generated by ColabFold-based Alpha Fold 2.0 was refined by a 3D refine server and a Galaxy refinement server (**Fig 6A**). The best refined structure showed a MoLProbity score of 1.284, a GDT-HA score of 0.9459, an RMSD score of 0.428, a poor rotamers score of 0.0, and a clash score of 5.3. The overall quality factor of the structure predicted by ERRAT was 99.351 **(Fig 6B).** The Ramachandran plot designed by Procheck showed 98.6% sequence in the most favored region **(Fig 6C**).

**Table 3:** Physicochemical properties of the vaccine.

Features	Properties	Assessment
Number of amino acids	171	Suitable
Total number of atoms	2392	-
Molecular weight	17543.29	Suitable
Theoretical pI	8.2	Basic
Total number of negatively charged residues (Asp + Glu)	8	-
Total number of positively charged residues (Arg + Lys)	10	-
Instability index (II)	22.44	Stable
Aliphatic index (AI)	63.16	Thermostable
Grand average of hydropathicity (GRAVY)	-0.32	Hydrophilic
Antigenicity	0.8028 (VaxiJen) 0.871809 (Antigen pro)	Antigenic
Allergenicity	Probable non allergen (AllerTop 2.0)	Non allergic
Toxicity	Non toxin (ToxinPred)	Non toxic

### 3.9. Molecular docking of vaccine with TLR4 receptor

**Fig 7:**
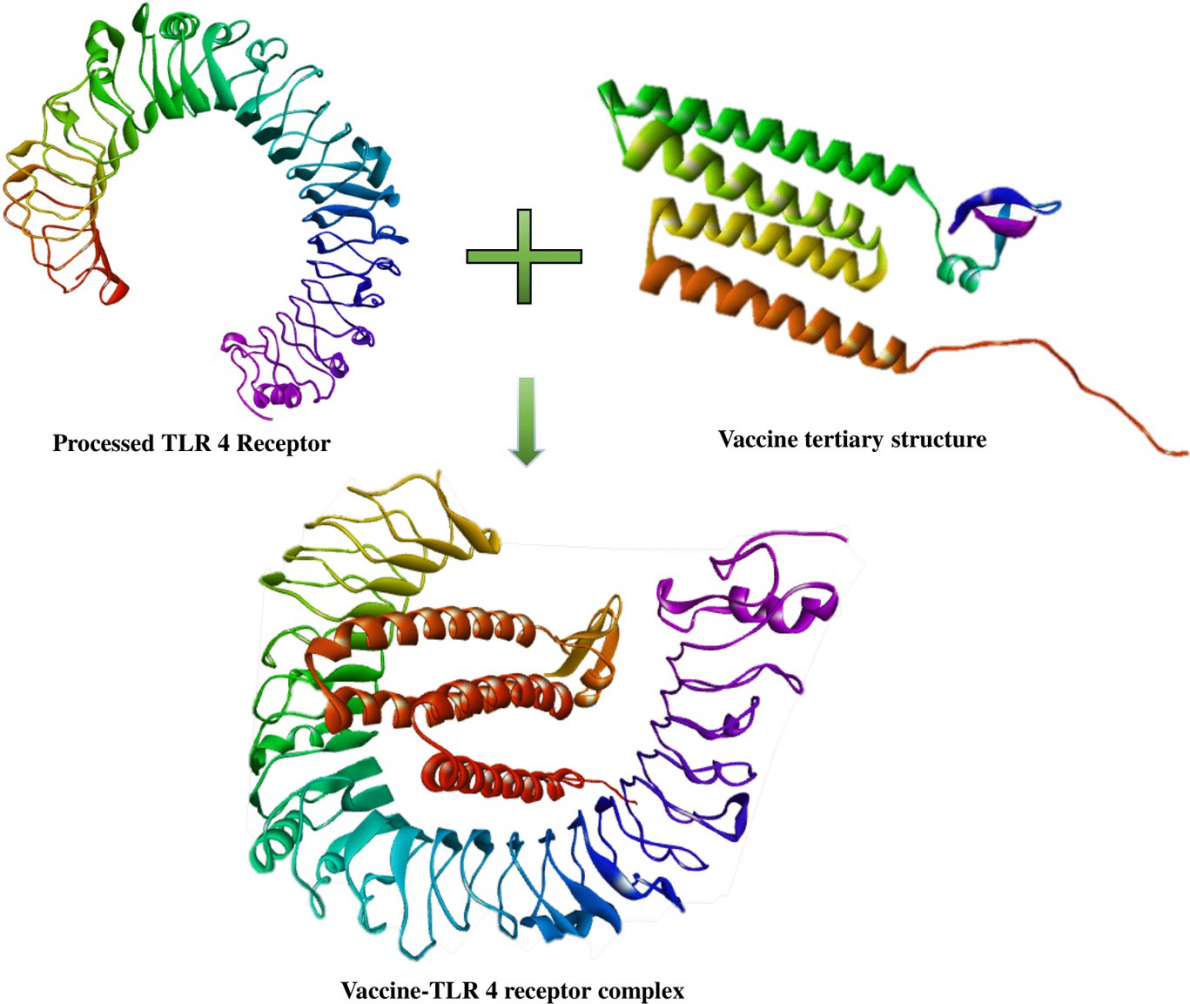
Vaccine-Toll Like Receptor 4 (TLR 4) complex.

Galaxy Tong Dock A produced 50 models for the vaccine-TLR4 receptor complex. Among the predicted models, model 1 was selected as the best-docked complex with a docking score of 1334.791 and a cluster size of 12 (**Fig 7**).

### 3.10. Immune simulation study

**Fig 8:**
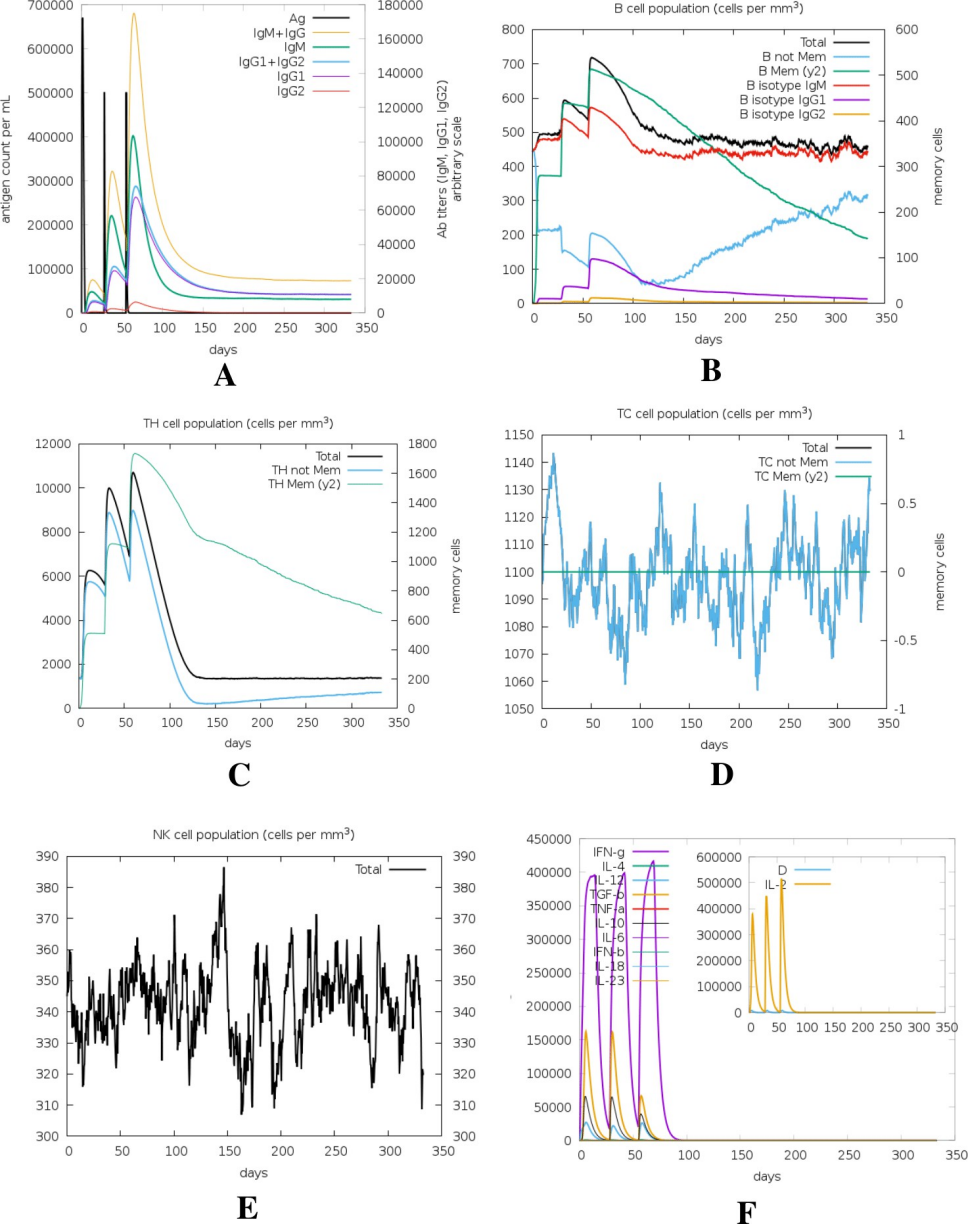
Immune simulation of the proposed vaccine. Here, (A) Antigen and immunoglobulin profiles, (B) B lymphocytes total count, memory cells, and subdivided in isotypes IgM, IgG1 and IgG2, (C) CD4 T-helper lymphocytes count, (D) CD8 T-cytotoxic lymphocytes count, (E) Natural Killer cells (total count), (F) Concentration of cytokines and interleukins.

The results from the *in silico* immune response simulation using the C-ImmSim server demonstrate a substantial increase in both the secondary and tertiary responses compared to the primary response. Notably, the second and third inoculation exhibited elevated levels of immunoglobulin action, including IgG1 + IgG2, IgM, and IgG + IgM antibodies, while concurrently showing a decrease in antigen concentration (**Fig 8A**). The simulation also revealed the presence of multiple B cell isotypes with long-term activity, suggesting potential isotype-switching abilities and memory formation **(Fig 8B**). In the same way, it was shown that the T helper and Cytotoxic T cell populations exhibited an increased response accompanied by the formation of relative memory (**Fig 8C and 8D**), a process that has significant importance in reinforcing the immune response. Additionally, the simulation showed heightened activity of natural killer cells **(Fig 8E**), and notable levels of IFN-γ and IL-2, indicative of a robust immune response. Importantly, a lower Simpson index (D) was observed, implying greater diversity in the immune response (**Fig 8F**).

### 3.11. The proposed vaccine altered TLR4 structure due to the interactions

**Fig 9:**
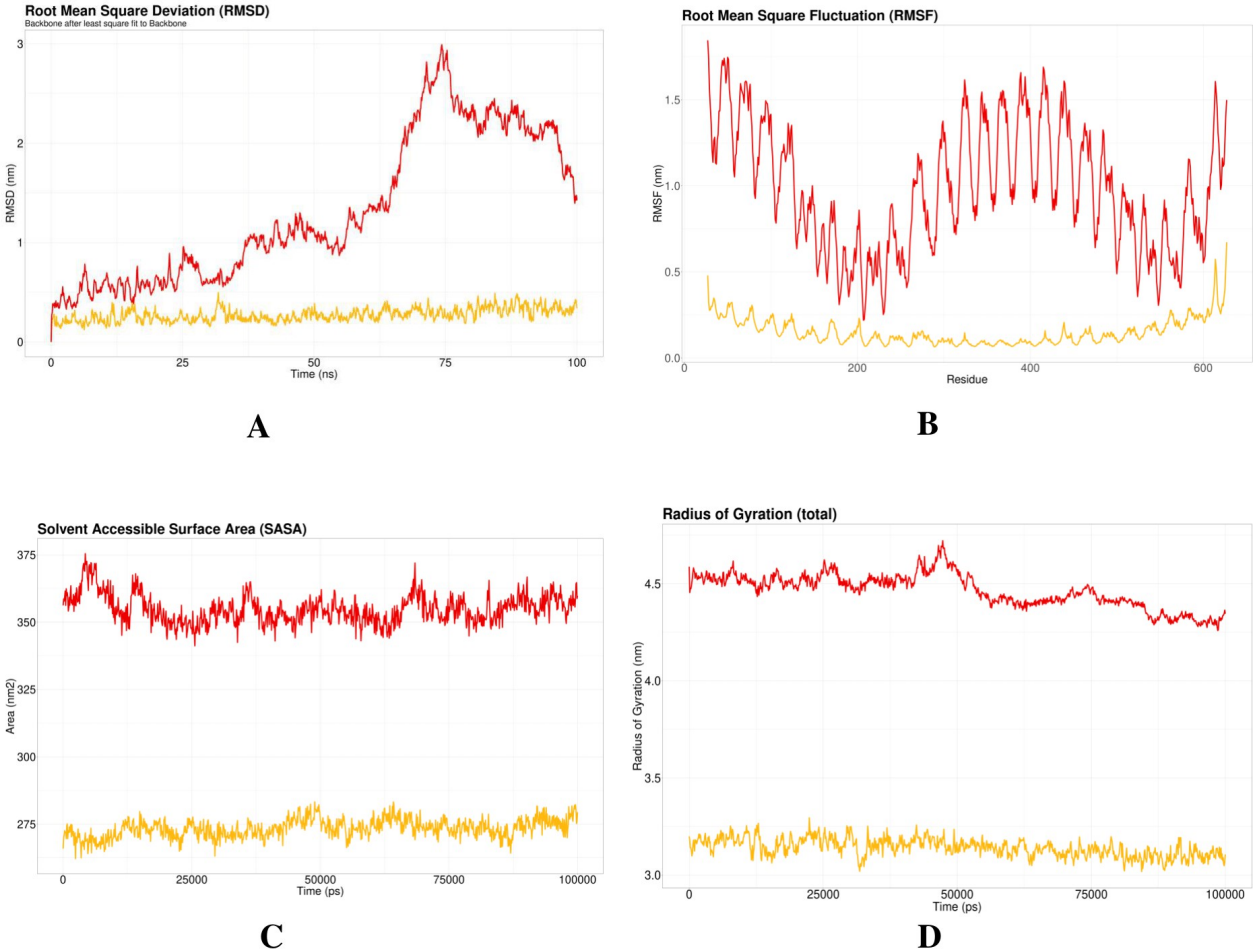
Molecular dynamic simulation where (A) represents Root Mean Square Deviation (RMSD), (B) demonstrates the Root Means Square Fluctuation (RMSF) profile of the protein, (C) Solvent Accessible Surface Area (SASA), and (D) depicted Radius of Gyration (Rg) result.

To evaluate the vaccine-TLR4 complex, we conducted a molecular dynamic simulation of only TLR4 (apo) (Fig 9: yellow lines) and TLR4-vaccine complex (Fig 9: red lines). After the simulation, the results were compared. To evaluate the conformational changes, the Root Mean Square Deviation (RMSD) was calculated. A significant change in the RMSD value corresponds to structural alterations due to ligand binding. In **Fig 9A**, the yellow line represents the RMSD profile of the apo-receptor or receptor complex, while the red line represents the vaccine-TLR4 complex. The RMSD value of the vaccine-TLR4 complex was finally ∼3 nm whereas the apo-receptor was ∼0.2 nm. A significant conformational difference was observed after 30 ns and 60 ns (**Fig 9A**). Root Mean Square Fluctuation (RMSF) depicts the mobility of the proteins with and without the presence of the selected vaccine. A higher RMSF indicates greater flexibility of a given amino acid position. **Fig 9B** demonstrates the RMSF profile of the proteins. The RMSF peaks were observed at ∼350^th^ residue, ∼400^th^ residue, and ∼600^th^ residue for the vaccine-TLR4 complex whereas the apo-receptor showed very low mobility.

### 3.12. Codon adaptation and visualization of cloning

**Fig 10:**
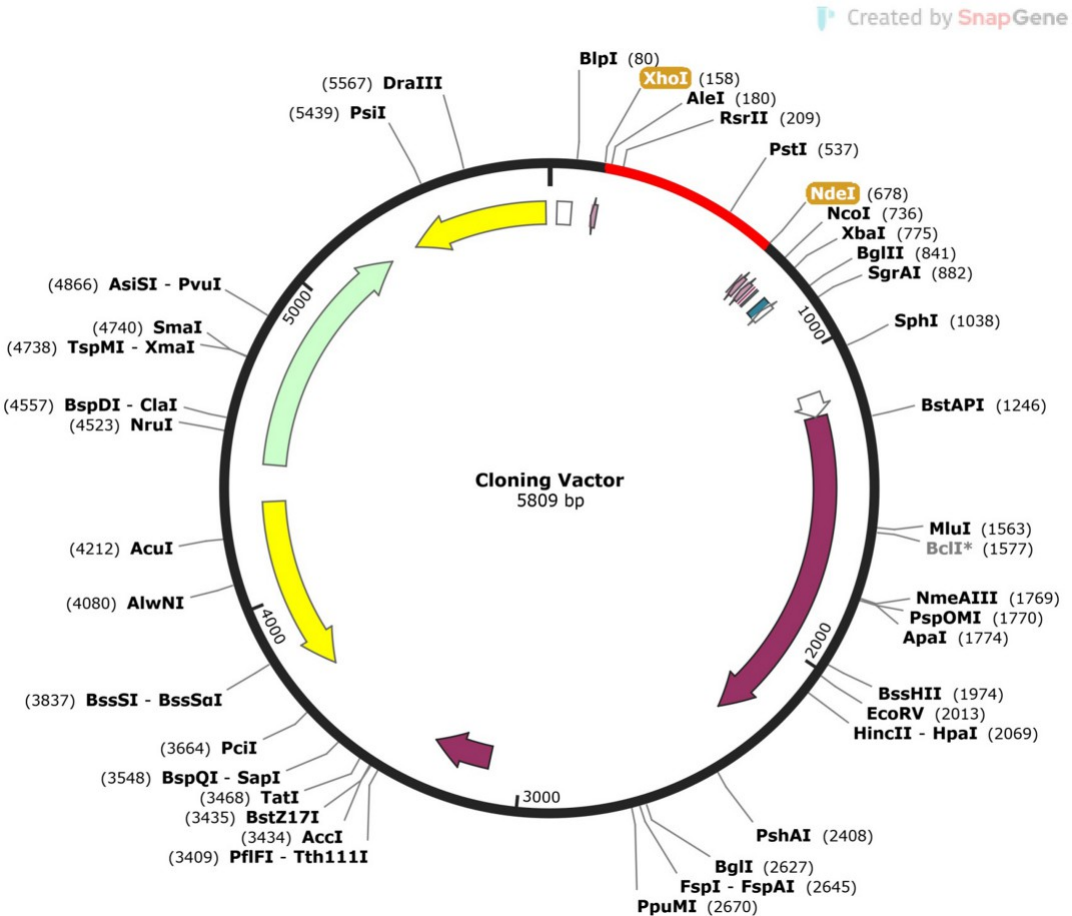
*In silico* cloning of the vaccine construct into the *E*. *coli* pET28a (+) plasmid vector. The red segment represents the vaccine sequence, flanked by XhoI and NdeI restriction sites.

The DNA sequence that was optimized had a length of 513 base pairs and a Codon Adaptation Index (CAI) value of 1.0. The GC content of the improved sequence was 54.97%. Flanked by restriction sites XhoI and NdeI, the sequence was introduced into the *E*. *coli* pET28a (+) Plasmid Vector via SnapGene tools. The final length of the cloning vector was 5.8 kb (**Fig 10**).

## 4. Discussion

*Salmonella* infections are a major cause of illness and mortality in low-resource environments, particularly in the Indian and Asian subcontinents and several regions of sub-Saharan Africa. More than 2500 *Salmonella* serotypes have been discovered, and the majority of the infection is caused by *Salmonella enterica* subsp. *enterica* [56]. In South Asia, around seven million people are affected every year resulting in 75,000 deaths [57]. The substantial morbidity and mortality caused by *Salmonella*, as well as the rising prevalence of antibiotic-resistant strains, have prompted the development of vaccinations against these organisms.

Currently, the only licensed *Salmonella* vaccine is the live-attenuated Ty21a vaccine. This vaccine offers protection against *S*. Typhi and provides limited cross-protection against *S*. Paratyphi B, although it does not extend to *S*. Paratyphi A. On the other hand, the injectable Vi capsular polysaccharide and conjugate vaccines effectively protect against *S*. Typhi infection but do not confer any cross-protection against other serovars of *Salmonella* [58]. The limited cross-reactivity and inadequate efficacy of existing vaccines against mutant viral strains underscore the urgency for the development of a novel and effective vaccine. Currently, there exists a diverse range of methodologies for the production and manufacturing of effective epitope-based vaccines [59].

In this study, we implemented comparative genomics and subtractive proteomics to analyze the core proteome of *Salmonella* species, aiming to design a multi-epitope vaccine. Multi-epitope-based vaccines were preferred over conventional vaccines due to their cost-effectiveness, superior safety, and the ability to tailor epitopes intelligently for increased potency [60]. Several previous studies have employed similar approaches to design multi-epitope vaccines; however, unlike our present studies, they targeted a particular strain of *Salmonella* [61,62]. Here, to develop a pan-*Salmonella* vaccine, we targeted the core proteome of *Salmonella* to design a vaccine that will be effective against a broad spectrum of *Salmonella* infections.

Initially, we executed comparative genomics on 30 *Salmonella* strains to find the core proteome of *Salmonella* spp. Afterward, subtractive proteomics filters were applied to that core proteome to select a suitable vaccine candidate. Among the 2395 core protein sequences obtained from 30 different *Salmonella* species, CsgA was finally elected. Plausibly, CsgA is not homologous to humans, and it is essential for the survival of the bacteria. Subcellular localization predicted that it is an extracellular protein (**S3 Table)**. In the quest for an ideal vaccine candidate, it is crucial to ensure that the selected protein is not present in the human gut microbiota. Therefore, based on the results, extracellular CsgA should be a prospective candidate [63]. A previous study reported that *Vibrio parahaemolyticus* CsgA can produce a robust immune response and protection against *V*. *parahaemolyticus* in BALB/c mice [55]. Moreover, *Salmonella* CsgA is highly conserved across many strains **(S1 Fig**), therefore, we selected CsgA as a vaccine candidate to develop a Pan-*Salmonella* vaccine.

The establishment of a prolonged and robust immune response necessitates the cooperative interaction between B cells and T cells, which is facilitated by cellular and humoral immunity. Hence, the B cells and T cell epitope of the vaccine candidate were predicted and assessed (**S4 Table**). The selection of epitopes for vaccine production involved the identification of two B cell epitopes and seven T cell epitopes **(Table 1**). The population coverage of our selected T cell epitopes was 98.17% (**Fig 4**), indicating a high level of effectiveness in reducing the transmission of *Salmonella* infections [64]. Additionally, molecular docking analysis predicted strong binding between the T cell epitopes and their corresponding MHC molecules (**Fig 3**) which also confirms the previous statement.

We have constructed the multi-epitope vaccine by joining the selected epitopes with suitable GPGPG and AYY linkers (**Fig 5**). These linkers improve the vaccine’s stability, folding, and expression [65]. To activate TLR4, we added the chemoattractant HBD-2 as an adjuvant at the end of the sequence using EAAAK linkers. HBD-2 adjuvant will enhance immune response and bind with TLR4 to activate specific pathways [66,67]. The physicochemical analysis of the vaccine design revealed that the vaccine carried a molecular weight of 17543.29 Da (**Table 3**). The vaccine exhibits a basic nature as indicated by its isoelectric point (pI) and instability index (II). Additionally, it demonstrates stability in E. coli, with a half-life above 10 hours. The vaccine is hydrophilic since the GRAVY value is just -0.320. These features are very important for separating the vaccine after it has been made in the E. coli pET28a (+) Plasmid Vector. Protein expression was optimized with a GC content of 54.97% and a Codon Adaptation Index (CAI) of 1.00% **(Fig 10)**.

The vaccine showed high antigenicity with no toxicity or allergenicity **(Table 3)**. The 3D structure of the vaccine is generated and validated (**Fig 6**). Ramachandran’s plot showed 98.6% residues in the most favored region, and an ERRAT value of 99.351 was found, eliciting the overall quality of the structure. A stable and substantial immune response depends on the binding ability of the vaccine with the host receptor [68]. Results from molecular docking and molecular dynamics simulation of vaccine-TLR4 suggested that the vaccine can bind and stabilize pattern recognition receptor (PRR) TLR4 in dynamic conditions with significant conformational changes **(Fig 9)**. These 3D interactions and dynamic properties suggest that the vaccine is capable of triggering innate immunity. To predict the responses from adaptive immunity *in silico* immune simulation was conducted. The immune simulation was conducted to replicate the real-life scenario of administering three vaccine injections, each containing 1000 units, with a four-week interval between them. By setting the time-step to 1050, which is equivalent to 8 hours in real life, the simulation aimed to accurately capture the dynamics of the vaccination process. The *in silico* immune response simulation using the C-ImmSim server shows (**Fig 8**) a significant increase in secondary and tertiary responses compared to the primary response. Secondary responses show higher levels of immunoglobulin action while reducing antigen concentration. Multiple B-cell isotypes are present, suggesting potential isotype-switching abilities and memory formation **(Fig 8A and 8B)**. T helper and Cytotoxic T cell populations also show an elevated response, reinforcing the immune response **(Fig 8C and 8D)**. Natural killer cells are heightened, and IFN-γ and IL-2 levels are observed (**Fig 8E and 8F)**. The smaller D value indicates a specific immune response [69]. Lastly, the efficiency and safety of the vaccine need to be determined through *in vivo*, *in vitro*, and clinical trial investigations.

## 5. Conclusion

In this study, an immunodominant vaccine that can protect against a variety of *Salmonella* strains has been proposed. Following appropriate clinical assessments, this vaccination can lower Salmonellosis rates globally.

## Supporting information

S1 Fig CsgA conservancy analysis result. (PNG)

S1 Table List of Serovar, Gene bank ID, Source, and Country. (DOCX)

S2 Table List of all the core proteins. (XLSX)

S3 Table Subcellular localization results. (TXT)

S4 Table List of all the predicted epitopes within CsgA. (DOCX)

S5 Table Toxicity prediction result. (DOCX)
