# Supplementary figures and images for: An integrated comparative genomics, subtractive proteomics and immunoinformatics framework for the rational design of a Pan-Salmonella multi-epitope vaccine
Source: PLoS One. 2024 Jul 3;19(7):e0292413. doi: 10.1371/journal.pone.0292413 (PMC11221655; doi:10.1371/journal.pone.0292413)

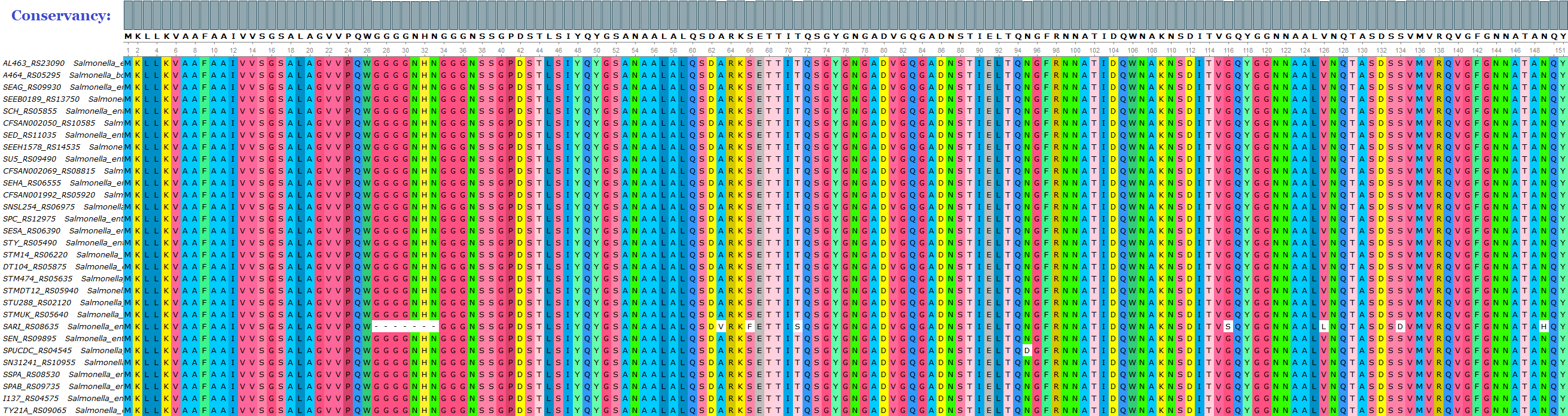

Supplement: S1 Fig — (PNG) [file pone.0292413.s001.png]
