# Supplementary material for: An integrated comparative genomics, subtractive proteomics and immunoinformatics framework for the rational design of a Pan-Salmonella multi-epitope vaccine
Source: PLoS One. 2024 Jul 3;19(7):e0292413. doi: 10.1371/journal.pone.0292413 (PMC11221655; doi:10.1371/journal.pone.0292413)
Supplement: S1 Table — (DOCX) [file pone.0292413.s002.docx]

| **Serovar** | **Gene bank ID** | **Source** | **Country** |
| --- | --- | --- | --- |
| ALL_Salmonella_enterica_strain_FDAARGOS_94_NZ_CP014051 | NZ_CP014051 | Unknown | USA |
| ALL_Salmonella_bongori_N268_08_NC_021870 | NC_021870 | Human | Switzerland |
| ALL_Salmonella_enterica_subsp_enterica_serovar_Agona_str_SL483_NC_011149 | NC_011149 | Unknown | USA |
| ALL_Salmonella_enterica_subsp_enterica_serovar_Bareilly_str_CFSAN000189_NC_021844 | NC_021844 | frozen raw shrimp | India |
| ALL_Salmonella_enterica_subsp_enterica_serovar_Choleraesuis_str_SC_B67_NC_006905 | NC_006905 | Human | Taiwan |
| ALL_Salmonella_enterica_subsp_enterica_serovar_Cubana_str_CFSAN002050_NC_021818 | NC_021818 | fresh alfalfa sprout | USA |
| ALL_Salmonella_enterica_subsp_enterica_serovar_Dublin_str_CT_02021853_NC_011205 | NC_011205 | Human | USA |
| ALL_Salmonella_enterica_subsp_enterica_serovar_Heidelberg_str_41578_NC_021810 | NC_021810 | Human stool | USA |
| ALL_Salmonella_enterica_subsp_enterica_serovar_Heidelberg_str_B182_NC_017623 | NC_017623 | Bovine feces | France |
| ALL_Salmonella_enterica_subsp_enterica_serovar_Heidelberg_str_CFSAN002069_NC_021812 | NC_021812 | chicken | USA |
| ALL_Salmonella_enterica_subsp_enterica_serovar_Heidelberg_str_SL476_NC_011083 | NC_011083 | Ground Turkey | USA |
| ALL_Salmonella_enterica_subsp_enterica_serovar_Javiana_str_CFSAN001992_NC_020307 | NC_020307 | Human | USA |
| ALL_Salmonella_enterica_subsp_enterica_serovar_Newport_str_SL254_NC_011080 | NC_011080 | Human | USA |
| ALL_Salmonella_enterica_subsp_enterica_serovar_Paratyphi_C_str_RKS4594_NC_012125 | NC_012125 | Human | China |
| ALL_Salmonella_enterica_subsp_enterica_serovar_Schwarzengrund_str_CVM19633_NC_011094 | NC_011094 | Human | USA |
| ALL_Salmonella_enterica_subsp_enterica_serovar_Typhi_str_CT18_NC_003198 | NC_003198 | Human | Vietnam |
| ALL_Salmonella_enterica_subsp_enterica_serovar_Typhimurium_str_14028S_NC_016856 | NC_016856 | Chicken | USA |
| ALL_Salmonella_enterica_subsp_enterica_serovar_Typhimurium_str_DT104_NC_022569 | NC_022569 | Animal | Scotland |
| ALL_Salmonella_enterica_subsp_enterica_serovar_Typhimurium_str_ST4_74_NC_016857 | NC_016857 | Calf | UK |
| ALL_Salmonella_enterica_subsp_enterica_serovar_Typhimurium_str_T000240_NC_016860 | NC_016860 | Human | Japan |
| ALL_Salmonella_enterica_subsp_enterica_serovar_Typhimurium_str_U288_NC_021151 | NC_021151 | Unknown | UK |
| ALL_Salmonella_enterica_subsp_enterica_serovar_Typhimurium_str_UK_1_NC_016863 | NC_016863 | Chik | USA |
| Salmonella_enterica_subsp_arizonae_serovar_62_z4z23__strain_RSK2980_NC_010067 | NC_010067 | Unknown | USA |
| Salmonella_enterica_subsp_enterica_serovar_Enteritidis_str_P125109_NC_011294 | NC_011294 | Chicken | UK |
| Salmonella_enterica_subsp_enterica_serovar_Gallinarum_pullorum_str_CDC1983_67_NC_022221 | NC_022221 | Unknown | China |
| Salmonella_enterica_subsp_enterica_serovar_Newport_str_USMARC_S3124_1_NC_021902 | NC_021902 | Cattle | USA |
| Salmonella_enterica_subsp_enterica_serovar_Paratyphi_A_str_AKU_12601_NC_011147 | NC_011147 | Human | UK |
| Salmonella_enterica_subsp_enterica_serovar_Paratyphi_B_str_SPB7_NC_010102 | NC_010102 | Human Stool | Malaysia |
| Salmonella_enterica_subsp_enterica_serovar_Pullorum_str_S06004_NC_021984 | NC_021984 | Unknown | China |
| Salmonella_enterica_subsp_enterica_serovar_Typhi_str_Ty21a_NC_021176 | NC_021176 | Human | USA |
