# Supplementary material for: An integrated comparative genomics, subtractive proteomics and immunoinformatics framework for the rational design of a Pan-Salmonella multi-epitope vaccine
Source: PLoS One. 2024 Jul 3;19(7):e0292413. doi: 10.1371/journal.pone.0292413 (PMC11221655; doi:10.1371/journal.pone.0292413)
Supplement: S4 Table — (DOCX) [file pone.0292413.s005.docx]

| **B Cell epitopes** | **Epitopes sequence** | | **Position** |
| --- | --- | --- | --- |
|  | GGGNHNGGGNSSGPD | | 2-16 |
|  | TQNGFRNN | | 67-74 |
|  | DQWNAKNSD | | 78-86 |
|  | NQTASDSS | | 101-108 |
| **CTL epitopes** | **Serotyp**e | **Epitope Sequence** | **Combined Score** |
|  | A1 | NSDITVGQY, ETTITQSGY, DSTLSIYQY, GADNSTIEL | 3.4692 |
|  | A2 | GADNSTIEL | 0.6175 |
|  | A3 | ATIDQWNAK | 0.7407 |
|  | A24 | QYGSANAAL, QYGGNNAAL | 1.0441 |
|  | A26 | ETTITQSGY, DSTLSIYQY, ATIDQWNAK | 2.2572 |
|  | B7 | GSANAALAL | 0.6405 |
|  | B8 | SVMVRQVGF | 0.5851 |
|  | B27 | FRNNATIDQ | 0.7674 |
|  | B39 | QYGGNNAAL, GADNSTIEL, GSANAALAL, YQYGSANAA, QYGSANAAL | 1.3689 |
|  | B44 | GSANAALAL | 0.6102 |
|  | B58 | RNNATIDQW, GSANAALAL | 1.3572 |
|  | B62 | SVMVRQVGF, YQYGSANAA, GQYGGNNAA, GNNATANQY, RQVGFGNNA, GSANAALAL | 1.1152 |
| **HTL epitopes** | **Allele name** | | **Sequence** |
|  | DRB1_0101 | | LSIYQYGSANAALAL (sb) |
|  | HLA-DPA10103-DPA10401 | | SVMVRQVGFGNNATA (wb) |
|  | HLA-DQA10201-DQB10301 | | SIYQYGSANAALALQ (sb) |
