# Supplementary material for: An integrated comparative genomics, subtractive proteomics and immunoinformatics framework for the rational design of a Pan-Salmonella multi-epitope vaccine
Source: PLoS One. 2024 Jul 3;19(7):e0292413. doi: 10.1371/journal.pone.0292413 (PMC11221655; doi:10.1371/journal.pone.0292413)
Supplement: S5 Table — (DOCX) [file pone.0292413.s006.docx]

| **Predicted peptide** | Toxicity [Prediction](https://webs.iiitd.edu.in/raghava/toxinpred/prot_submitfreq_S.php?ran=6491) | Predicted peptide | Toxicity [Prediction](https://webs.iiitd.edu.in/raghava/toxinpred/prot_submitfreq_S.php?ran=6491) | Predicted peptide | Toxicity [Prediction](https://webs.iiitd.edu.in/raghava/toxinpred/prot_submitfreq_S.php?ran=6491) |
| --- | --- | --- | --- | --- | --- |
| [GGGNHNGGGN](https://webs.iiitd.edu.in/raghava/toxinpred/pepsearch_S.php?seq=GGGNHNGGGN&thval=0.0) | Non-Toxin | [AAYQYGSANA](https://webs.iiitd.edu.in/raghava/toxinpred/pepsearch_S.php?seq=AAYQYGSANA&thval=0.0) | Non-Toxin | [NATIDQWAAY](https://webs.iiitd.edu.in/raghava/toxinpred/pepsearch_S.php?seq=NATIDQWAAY&thval=0.0) | Non-Toxin |
| [GGNHNGGGNS](https://webs.iiitd.edu.in/raghava/toxinpred/pepsearch_S.php?seq=GGNHNGGGNS&thval=0.0) | Non-Toxin | [AYQYGSANAA](https://webs.iiitd.edu.in/raghava/toxinpred/pepsearch_S.php?seq=AYQYGSANAA&thval=0.0) | Non-Toxin | [ATIDQWAAYL](https://webs.iiitd.edu.in/raghava/toxinpred/pepsearch_S.php?seq=ATIDQWAAYL&thval=0.0) | Non-Toxin |
| [GNHNGGGNSS](https://webs.iiitd.edu.in/raghava/toxinpred/pepsearch_S.php?seq=GNHNGGGNSS&thval=0.0) | Non-Toxin | [YQYGSANAAL](https://webs.iiitd.edu.in/raghava/toxinpred/pepsearch_S.php?seq=YQYGSANAAL&thval=0.0) | Non-Toxin | [TIDQWAAYLS](https://webs.iiitd.edu.in/raghava/toxinpred/pepsearch_S.php?seq=TIDQWAAYLS&thval=0.0) | Non-Toxin |
| [NHNGGGNSSG](https://webs.iiitd.edu.in/raghava/toxinpred/pepsearch_S.php?seq=NHNGGGNSSG&thval=0.0) | Non-Toxin | [QYGSANAALA](https://webs.iiitd.edu.in/raghava/toxinpred/pepsearch_S.php?seq=QYGSANAALA&thval=0.0) | Non-Toxin | [IDQWAAYLSI](https://webs.iiitd.edu.in/raghava/toxinpred/pepsearch_S.php?seq=IDQWAAYLSI&thval=0.0) | Non-Toxin |
| [HNGGGNSSGP](https://webs.iiitd.edu.in/raghava/toxinpred/pepsearch_S.php?seq=HNGGGNSSGP&thval=0.0) | Non-Toxin | [YGSANAALAA](https://webs.iiitd.edu.in/raghava/toxinpred/pepsearch_S.php?seq=YGSANAALAA&thval=0.0) | Non-Toxin | [DQWAAYLSIY](https://webs.iiitd.edu.in/raghava/toxinpred/pepsearch_S.php?seq=DQWAAYLSIY&thval=0.0) | Non-Toxin |
| [NGGGNSSGPD](https://webs.iiitd.edu.in/raghava/toxinpred/pepsearch_S.php?seq=NGGGNSSGPD&thval=0.0) | Non-Toxin | [GSANAALAAY](https://webs.iiitd.edu.in/raghava/toxinpred/pepsearch_S.php?seq=GSANAALAAY&thval=0.0) | Non-Toxin | [QWAAYLSIYQ](https://webs.iiitd.edu.in/raghava/toxinpred/pepsearch_S.php?seq=QWAAYLSIYQ&thval=0.0) | Non-Toxin |
| [GGGNSSGPDG](https://webs.iiitd.edu.in/raghava/toxinpred/pepsearch_S.php?seq=GGGNSSGPDG&thval=0.0) | Non-Toxin | [SANAALAAYE](https://webs.iiitd.edu.in/raghava/toxinpred/pepsearch_S.php?seq=SANAALAAYE&thval=0.0) | Non-Toxin | [WAAYLSIYQY](https://webs.iiitd.edu.in/raghava/toxinpred/pepsearch_S.php?seq=WAAYLSIYQY&thval=0.0) | Non-Toxin |
| [GGNSSGPDGP](https://webs.iiitd.edu.in/raghava/toxinpred/pepsearch_S.php?seq=GGNSSGPDGP&thval=0.0) | Non-Toxin | [ANAALAAYET](https://webs.iiitd.edu.in/raghava/toxinpred/pepsearch_S.php?seq=ANAALAAYET&thval=0.0) | Non-Toxin | [AAYLSIYQYG](https://webs.iiitd.edu.in/raghava/toxinpred/pepsearch_S.php?seq=AAYLSIYQYG&thval=0.0) | Non-Toxin |
| [GNSSGPDGPG](https://webs.iiitd.edu.in/raghava/toxinpred/pepsearch_S.php?seq=GNSSGPDGPG&thval=0.0) | Non-Toxin | [NAALAAYETT](https://webs.iiitd.edu.in/raghava/toxinpred/pepsearch_S.php?seq=NAALAAYETT&thval=0.0) | Non-Toxin | [AYLSIYQYGS](https://webs.iiitd.edu.in/raghava/toxinpred/pepsearch_S.php?seq=AYLSIYQYGS&thval=0.0) | Non-Toxin |
| [NSSGPDGPGP](https://webs.iiitd.edu.in/raghava/toxinpred/pepsearch_S.php?seq=NSSGPDGPGP&thval=0.0) | Non-Toxin | [AALAAYETTI](https://webs.iiitd.edu.in/raghava/toxinpred/pepsearch_S.php?seq=AALAAYETTI&thval=0.0) | Non-Toxin | [YLSIYQYGSA](https://webs.iiitd.edu.in/raghava/toxinpred/pepsearch_S.php?seq=YLSIYQYGSA&thval=0.0) | Non-Toxin |
| [SSGPDGPGPG](https://webs.iiitd.edu.in/raghava/toxinpred/pepsearch_S.php?seq=SSGPDGPGPG&thval=0.0) | Non-Toxin | [ALAAYETTIT](https://webs.iiitd.edu.in/raghava/toxinpred/pepsearch_S.php?seq=ALAAYETTIT&thval=0.0) | Non-Toxin | [LSIYQYGSAN](https://webs.iiitd.edu.in/raghava/toxinpred/pepsearch_S.php?seq=LSIYQYGSAN&thval=0.0) | Non-Toxin |
| [SGPDGPGPGD](https://webs.iiitd.edu.in/raghava/toxinpred/pepsearch_S.php?seq=SGPDGPGPGD&thval=0.0) | Non-Toxin | [LAAYETTITQ](https://webs.iiitd.edu.in/raghava/toxinpred/pepsearch_S.php?seq=LAAYETTITQ&thval=0.0) | Non-Toxin | [SIYQYGSANA](https://webs.iiitd.edu.in/raghava/toxinpred/pepsearch_S.php?seq=SIYQYGSANA&thval=0.0) | Non-Toxin |
| [GPDGPGPGDQ](https://webs.iiitd.edu.in/raghava/toxinpred/pepsearch_S.php?seq=GPDGPGPGDQ&thval=0.0) | Non-Toxin | [AAYETTITQS](https://webs.iiitd.edu.in/raghava/toxinpred/pepsearch_S.php?seq=AAYETTITQS&thval=0.0) | Non-Toxin | [IYQYGSANAA](https://webs.iiitd.edu.in/raghava/toxinpred/pepsearch_S.php?seq=IYQYGSANAA&thval=0.0) | Non-Toxin |
| [PDGPGPGDQW](https://webs.iiitd.edu.in/raghava/toxinpred/pepsearch_S.php?seq=PDGPGPGDQW&thval=0.0) | Non-Toxin | [AYETTITQSG](https://webs.iiitd.edu.in/raghava/toxinpred/pepsearch_S.php?seq=AYETTITQSG&thval=0.0) | Non-Toxin | [YQYGSANAAL](https://webs.iiitd.edu.in/raghava/toxinpred/pepsearch_S.php?seq=YQYGSANAAL&thval=0.0) | Non-Toxin |
| [DGPGPGDQWN](https://webs.iiitd.edu.in/raghava/toxinpred/pepsearch_S.php?seq=DGPGPGDQWN&thval=0.0) | Non-Toxin | [YETTITQSGY](https://webs.iiitd.edu.in/raghava/toxinpred/pepsearch_S.php?seq=YETTITQSGY&thval=0.0) | Non-Toxin | [QYGSANAALA](https://webs.iiitd.edu.in/raghava/toxinpred/pepsearch_S.php?seq=QYGSANAALA&thval=0.0) | Non-Toxin |
| [GPGPGDQWNA](https://webs.iiitd.edu.in/raghava/toxinpred/pepsearch_S.php?seq=GPGPGDQWNA&thval=0.0) | Non-Toxin | [ETTITQSGYA](https://webs.iiitd.edu.in/raghava/toxinpred/pepsearch_S.php?seq=ETTITQSGYA&thval=0.0) | Non-Toxin | [YGSANAALAL](https://webs.iiitd.edu.in/raghava/toxinpred/pepsearch_S.php?seq=YGSANAALAL&thval=0.0) | Non-Toxin |
| [PGPGDQWNAK](https://webs.iiitd.edu.in/raghava/toxinpred/pepsearch_S.php?seq=PGPGDQWNAK&thval=0.0) | Non-Toxin | [TTITQSGYAA](https://webs.iiitd.edu.in/raghava/toxinpred/pepsearch_S.php?seq=TTITQSGYAA&thval=0.0) | Non-Toxin | [GSANAALALA](https://webs.iiitd.edu.in/raghava/toxinpred/pepsearch_S.php?seq=GSANAALALA&thval=0.0) | Non-Toxin |
| [GPGDQWNAKN](https://webs.iiitd.edu.in/raghava/toxinpred/pepsearch_S.php?seq=GPGDQWNAKN&thval=0.0) | Non-Toxin | [TITQSGYAAY](https://webs.iiitd.edu.in/raghava/toxinpred/pepsearch_S.php?seq=TITQSGYAAY&thval=0.0) | Non-Toxin | [SANAALALAA](https://webs.iiitd.edu.in/raghava/toxinpred/pepsearch_S.php?seq=SANAALALAA&thval=0.0) | Non-Toxin |
| [PGDQWNAKNS](https://webs.iiitd.edu.in/raghava/toxinpred/pepsearch_S.php?seq=PGDQWNAKNS&thval=0.0) | Non-Toxin | [ITQSGYAAYQ](https://webs.iiitd.edu.in/raghava/toxinpred/pepsearch_S.php?seq=ITQSGYAAYQ&thval=0.0) | Non-Toxin | [ANAALALAAY](https://webs.iiitd.edu.in/raghava/toxinpred/pepsearch_S.php?seq=ANAALALAAY&thval=0.0) | Non-Toxin |
| [GDQWNAKNSD](https://webs.iiitd.edu.in/raghava/toxinpred/pepsearch_S.php?seq=GDQWNAKNSD&thval=0.0) | Non-Toxin | [TQSGYAAYQY](https://webs.iiitd.edu.in/raghava/toxinpred/pepsearch_S.php?seq=TQSGYAAYQY&thval=0.0) | Non-Toxin | [NAALALAAYS](https://webs.iiitd.edu.in/raghava/toxinpred/pepsearch_S.php?seq=NAALALAAYS&thval=0.0) | Non-Toxin |
| [DQWNAKNSDA](https://webs.iiitd.edu.in/raghava/toxinpred/pepsearch_S.php?seq=DQWNAKNSDA&thval=0.0) | Non-Toxin | [QSGYAAYQYG](https://webs.iiitd.edu.in/raghava/toxinpred/pepsearch_S.php?seq=QSGYAAYQYG&thval=0.0) | Non-Toxin | [AALALAAYSI](https://webs.iiitd.edu.in/raghava/toxinpred/pepsearch_S.php?seq=AALALAAYSI&thval=0.0) | Non-Toxin |
| [QWNAKNSDAA](https://webs.iiitd.edu.in/raghava/toxinpred/pepsearch_S.php?seq=QWNAKNSDAA&thval=0.0) | Non-Toxin | [SGYAAYQYGG](https://webs.iiitd.edu.in/raghava/toxinpred/pepsearch_S.php?seq=SGYAAYQYGG&thval=0.0) | Non-Toxin | [ALALAAYSIY](https://webs.iiitd.edu.in/raghava/toxinpred/pepsearch_S.php?seq=ALALAAYSIY&thval=0.0) | Non-Toxin |
| [WNAKNSDAAY](https://webs.iiitd.edu.in/raghava/toxinpred/pepsearch_S.php?seq=WNAKNSDAAY&thval=0.0) | Non-Toxin | [GYAAYQYGGN](https://webs.iiitd.edu.in/raghava/toxinpred/pepsearch_S.php?seq=GYAAYQYGGN&thval=0.0) | Non-Toxin | [LALAAYSIYQ](https://webs.iiitd.edu.in/raghava/toxinpred/pepsearch_S.php?seq=LALAAYSIYQ&thval=0.0) | Non-Toxin |
| [NAKNSDAAYN](https://webs.iiitd.edu.in/raghava/toxinpred/pepsearch_S.php?seq=NAKNSDAAYN&thval=0.0) | Non-Toxin | [YAAYQYGGNN](https://webs.iiitd.edu.in/raghava/toxinpred/pepsearch_S.php?seq=YAAYQYGGNN&thval=0.0) | Non-Toxin | [ALAAYSIYQY](https://webs.iiitd.edu.in/raghava/toxinpred/pepsearch_S.php?seq=ALAAYSIYQY&thval=0.0) | Non-Toxin |
| [AKNSDAAYNS](https://webs.iiitd.edu.in/raghava/toxinpred/pepsearch_S.php?seq=AKNSDAAYNS&thval=0.0) | Non-Toxin | [AAYQYGGNNA](https://webs.iiitd.edu.in/raghava/toxinpred/pepsearch_S.php?seq=AAYQYGGNNA&thval=0.0) | Non-Toxin | [LAAYSIYQYG](https://webs.iiitd.edu.in/raghava/toxinpred/pepsearch_S.php?seq=LAAYSIYQYG&thval=0.0) | Non-Toxin |
| [KNSDAAYNSD](https://webs.iiitd.edu.in/raghava/toxinpred/pepsearch_S.php?seq=KNSDAAYNSD&thval=0.0) | Non-Toxin | [AYQYGGNNAA](https://webs.iiitd.edu.in/raghava/toxinpred/pepsearch_S.php?seq=AYQYGGNNAA&thval=0.0) | Non-Toxin | [AAYSIYQYGS](https://webs.iiitd.edu.in/raghava/toxinpred/pepsearch_S.php?seq=AAYSIYQYGS&thval=0.0) | Non-Toxin |
| [NSDAAYNSDI](https://webs.iiitd.edu.in/raghava/toxinpred/pepsearch_S.php?seq=NSDAAYNSDI&thval=0.0) | Non-Toxin | [YQYGGNNAAL](https://webs.iiitd.edu.in/raghava/toxinpred/pepsearch_S.php?seq=YQYGGNNAAL&thval=0.0) | Non-Toxin | [AYSIYQYGSA](https://webs.iiitd.edu.in/raghava/toxinpred/pepsearch_S.php?seq=AYSIYQYGSA&thval=0.0) | Non-Toxin |
| [SDAAYNSDIT](https://webs.iiitd.edu.in/raghava/toxinpred/pepsearch_S.php?seq=SDAAYNSDIT&thval=0.0) | Non-Toxin | [QYGGNNAALA](https://webs.iiitd.edu.in/raghava/toxinpred/pepsearch_S.php?seq=QYGGNNAALA&thval=0.0) | Non-Toxin | [YSIYQYGSAN](https://webs.iiitd.edu.in/raghava/toxinpred/pepsearch_S.php?seq=YSIYQYGSAN&thval=0.0) | Non-Toxin |
| [DAAYNSDITV](https://webs.iiitd.edu.in/raghava/toxinpred/pepsearch_S.php?seq=DAAYNSDITV&thval=0.0) | Non-Toxin | [YGGNNAALAA](https://webs.iiitd.edu.in/raghava/toxinpred/pepsearch_S.php?seq=YGGNNAALAA&thval=0.0) | Non-Toxin | [SIYQYGSANA](https://webs.iiitd.edu.in/raghava/toxinpred/pepsearch_S.php?seq=SIYQYGSANA&thval=0.0) | Non-Toxin |
| [AAYNSDITVG](https://webs.iiitd.edu.in/raghava/toxinpred/pepsearch_S.php?seq=AAYNSDITVG&thval=0.0) | Non-Toxin | [GGNNAALAAY](https://webs.iiitd.edu.in/raghava/toxinpred/pepsearch_S.php?seq=GGNNAALAAY&thval=0.0) | Non-Toxin | [IYQYGSANAA](https://webs.iiitd.edu.in/raghava/toxinpred/pepsearch_S.php?seq=IYQYGSANAA&thval=0.0) | Non-Toxin |
| [AYNSDITVGQ](https://webs.iiitd.edu.in/raghava/toxinpred/pepsearch_S.php?seq=AYNSDITVGQ&thval=0.0) | Non-Toxin | [GNNAALAAYR](https://webs.iiitd.edu.in/raghava/toxinpred/pepsearch_S.php?seq=GNNAALAAYR&thval=0.0) | Non-Toxin | [YQYGSANAAL](https://webs.iiitd.edu.in/raghava/toxinpred/pepsearch_S.php?seq=YQYGSANAAL&thval=0.0) | Non-Toxin |
| [YNSDITVGQY](https://webs.iiitd.edu.in/raghava/toxinpred/pepsearch_S.php?seq=YNSDITVGQY&thval=0.0) | Non-Toxin | [NNAALAAYRN](https://webs.iiitd.edu.in/raghava/toxinpred/pepsearch_S.php?seq=NNAALAAYRN&thval=0.0) | Non-Toxin | [QYGSANAALA](https://webs.iiitd.edu.in/raghava/toxinpred/pepsearch_S.php?seq=QYGSANAALA&thval=0.0) | Non-Toxin |
| [NSDITVGQYA](https://webs.iiitd.edu.in/raghava/toxinpred/pepsearch_S.php?seq=NSDITVGQYA&thval=0.0) | Non-Toxin | [NAALAAYRNN](https://webs.iiitd.edu.in/raghava/toxinpred/pepsearch_S.php?seq=NAALAAYRNN&thval=0.0) | Non-Toxin | [YGSANAALAL](https://webs.iiitd.edu.in/raghava/toxinpred/pepsearch_S.php?seq=YGSANAALAL&thval=0.0) | Non-Toxin |
| [SDITVGQYAA](https://webs.iiitd.edu.in/raghava/toxinpred/pepsearch_S.php?seq=SDITVGQYAA&thval=0.0) | Non-Toxin | [AALAAYRNNA](https://webs.iiitd.edu.in/raghava/toxinpred/pepsearch_S.php?seq=AALAAYRNNA&thval=0.0) | Non-Toxin | [GSANAALALQ](https://webs.iiitd.edu.in/raghava/toxinpred/pepsearch_S.php?seq=GSANAALALQ&thval=0.0) | Non-Toxin |
| [DITVGQYAAY](https://webs.iiitd.edu.in/raghava/toxinpred/pepsearch_S.php?seq=DITVGQYAAY&thval=0.0) | Non-Toxin | [ALAAYRNNAT](https://webs.iiitd.edu.in/raghava/toxinpred/pepsearch_S.php?seq=ALAAYRNNAT&thval=0.0) | Non-Toxin | [SANAALALQE](https://webs.iiitd.edu.in/raghava/toxinpred/pepsearch_S.php?seq=SANAALALQE&thval=0.0) | Non-Toxin |
| [ITVGQYAAYQ](https://webs.iiitd.edu.in/raghava/toxinpred/pepsearch_S.php?seq=ITVGQYAAYQ&thval=0.0) | Non-Toxin | [LAAYRNNATI](https://webs.iiitd.edu.in/raghava/toxinpred/pepsearch_S.php?seq=LAAYRNNATI&thval=0.0) | Non-Toxin | [ANAALALQEA](https://webs.iiitd.edu.in/raghava/toxinpred/pepsearch_S.php?seq=ANAALALQEA&thval=0.0) | Non-Toxin |
| [TVGQYAAYQY](https://webs.iiitd.edu.in/raghava/toxinpred/pepsearch_S.php?seq=TVGQYAAYQY&thval=0.0) | Non-Toxin | [AAYRNNATID](https://webs.iiitd.edu.in/raghava/toxinpred/pepsearch_S.php?seq=AAYRNNATID&thval=0.0) | Non-Toxin | [NAALALQEAA](https://webs.iiitd.edu.in/raghava/toxinpred/pepsearch_S.php?seq=NAALALQEAA&thval=0.0) | Non-Toxin |
| [VGQYAAYQYG](https://webs.iiitd.edu.in/raghava/toxinpred/pepsearch_S.php?seq=VGQYAAYQYG&thval=0.0) | Non-Toxin | [AYRNNATIDQ](https://webs.iiitd.edu.in/raghava/toxinpred/pepsearch_S.php?seq=AYRNNATIDQ&thval=0.0) | Non-Toxin | [AALALQEAAA](https://webs.iiitd.edu.in/raghava/toxinpred/pepsearch_S.php?seq=AALALQEAAA&thval=0.0) | Non-Toxin |
| [GQYAAYQYGS](https://webs.iiitd.edu.in/raghava/toxinpred/pepsearch_S.php?seq=GQYAAYQYGS&thval=0.0) | Non-Toxin | [YRNNATIDQW](https://webs.iiitd.edu.in/raghava/toxinpred/pepsearch_S.php?seq=YRNNATIDQW&thval=0.0) | Non-Toxin | [ALALQEAAAK](https://webs.iiitd.edu.in/raghava/toxinpred/pepsearch_S.php?seq=ALALQEAAAK&thval=0.0) | Non-Toxin |
| [QYAAYQYGSA](https://webs.iiitd.edu.in/raghava/toxinpred/pepsearch_S.php?seq=QYAAYQYGSA&thval=0.0) | Non-Toxin | [RNNATIDQWA](https://webs.iiitd.edu.in/raghava/toxinpred/pepsearch_S.php?seq=RNNATIDQWA&thval=0.0) | Non-Toxin | [LALQEAAAKG](https://webs.iiitd.edu.in/raghava/toxinpred/pepsearch_S.php?seq=LALQEAAAKG&thval=0.0) | Non-Toxin |
| [YAAYQYGSAN](https://webs.iiitd.edu.in/raghava/toxinpred/pepsearch_S.php?seq=YAAYQYGSAN&thval=0.0) | Non-Toxin | [NNATIDQWAA](https://webs.iiitd.edu.in/raghava/toxinpred/pepsearch_S.php?seq=NNATIDQWAA&thval=0.0) | Non-Toxin | [ALQEAAAKGI](https://webs.iiitd.edu.in/raghava/toxinpred/pepsearch_S.php?seq=ALQEAAAKGI&thval=0.0) | Non-Toxin |
| [AAAKGIGDPV](https://webs.iiitd.edu.in/raghava/toxinpred/pepsearch_S.php?seq=AAAKGIGDPV&thval=0.0) | Non-Toxin | [ICHPVFCPRR](https://webs.iiitd.edu.in/raghava/toxinpred/pepsearch_S.php?seq=ICHPVFCPRR&thval=0.0) | Non-Toxin | [PRRYKQIGTC](https://webs.iiitd.edu.in/raghava/toxinpred/pepsearch_S.php?seq=PRRYKQIGTC&thval=0.0) | Non-Toxin |
| [AAKGIGDPVT](https://webs.iiitd.edu.in/raghava/toxinpred/pepsearch_S.php?seq=AAKGIGDPVT&thval=0.0) | Non-Toxin | [CHPVFCPRRY](https://webs.iiitd.edu.in/raghava/toxinpred/pepsearch_S.php?seq=CHPVFCPRRY&thval=0.0) | Non-Toxin | [RRYKQIGTCG](https://webs.iiitd.edu.in/raghava/toxinpred/pepsearch_S.php?seq=RRYKQIGTCG&thval=0.0) | Non-Toxin |
| [AKGIGDPVTC](https://webs.iiitd.edu.in/raghava/toxinpred/pepsearch_S.php?seq=AKGIGDPVTC&thval=0.0) | Non-Toxin | [HPVFCPRRYK](https://webs.iiitd.edu.in/raghava/toxinpred/pepsearch_S.php?seq=HPVFCPRRYK&thval=0.0) | Non-Toxin | [RYKQIGTCGL](https://webs.iiitd.edu.in/raghava/toxinpred/pepsearch_S.php?seq=RYKQIGTCGL&thval=0.0) | Non-Toxin |
| [KGIGDPVTCL](https://webs.iiitd.edu.in/raghava/toxinpred/pepsearch_S.php?seq=KGIGDPVTCL&thval=0.0) | Non-Toxin | [PVFCPRRYKQ](https://webs.iiitd.edu.in/raghava/toxinpred/pepsearch_S.php?seq=PVFCPRRYKQ&thval=0.0) | Non-Toxin | [YKQIGTCGLP](https://webs.iiitd.edu.in/raghava/toxinpred/pepsearch_S.php?seq=YKQIGTCGLP&thval=0.0) | Non-Toxin |
| [GIGDPVTCLK](https://webs.iiitd.edu.in/raghava/toxinpred/pepsearch_S.php?seq=GIGDPVTCLK&thval=0.0) | Non-Toxin | [VFCPRRYKQI](https://webs.iiitd.edu.in/raghava/toxinpred/pepsearch_S.php?seq=VFCPRRYKQI&thval=0.0) | Non-Toxin | [KQIGTCGLPG](https://webs.iiitd.edu.in/raghava/toxinpred/pepsearch_S.php?seq=KQIGTCGLPG&thval=0.0) | Non-Toxin |
| [IGDPVTCLKS](https://webs.iiitd.edu.in/raghava/toxinpred/pepsearch_S.php?seq=IGDPVTCLKS&thval=0.0) | Non-Toxin | [FCPRRYKQIG](https://webs.iiitd.edu.in/raghava/toxinpred/pepsearch_S.php?seq=FCPRRYKQIG&thval=0.0) | Non-Toxin | [QIGTCGLPGT](https://webs.iiitd.edu.in/raghava/toxinpred/pepsearch_S.php?seq=QIGTCGLPGT&thval=0.0) | Non-Toxin |
| [GDPVTCLKSG](https://webs.iiitd.edu.in/raghava/toxinpred/pepsearch_S.php?seq=GDPVTCLKSG&thval=0.0) | Non-Toxin | [CPRRYKQIGT](https://webs.iiitd.edu.in/raghava/toxinpred/pepsearch_S.php?seq=CPRRYKQIGT&thval=0.0) | Non-Toxin | [IGTCGLPGTK](https://webs.iiitd.edu.in/raghava/toxinpred/pepsearch_S.php?seq=IGTCGLPGTK&thval=0.0) | Non-Toxin |
| [DPVTCLKSGA](https://webs.iiitd.edu.in/raghava/toxinpred/pepsearch_S.php?seq=DPVTCLKSGA&thval=0.0) | Non-Toxin | [LQEAAAKGIG](https://webs.iiitd.edu.in/raghava/toxinpred/pepsearch_S.php?seq=LQEAAAKGIG&thval=0.0) | Non-Toxin | [GTCGLPGTKC](https://webs.iiitd.edu.in/raghava/toxinpred/pepsearch_S.php?seq=GTCGLPGTKC&thval=0.0) | Non-Toxin |
| [PVTCLKSGAI](https://webs.iiitd.edu.in/raghava/toxinpred/pepsearch_S.php?seq=PVTCLKSGAI&thval=0.0) | Non-Toxin | [QEAAAKGIGD](https://webs.iiitd.edu.in/raghava/toxinpred/pepsearch_S.php?seq=QEAAAKGIGD&thval=0.0) | Non-Toxin | [TCGLPGTKCC](https://webs.iiitd.edu.in/raghava/toxinpred/pepsearch_S.php?seq=TCGLPGTKCC&thval=0.0) | Toxin |
| [VTCLKSGAIC](https://webs.iiitd.edu.in/raghava/toxinpred/pepsearch_S.php?seq=VTCLKSGAIC&thval=0.0) | Non-Toxin | [EAAAKGIGDP](https://webs.iiitd.edu.in/raghava/toxinpred/pepsearch_S.php?seq=EAAAKGIGDP&thval=0.0) | Non-Toxin | [CGLPGTKCCK](https://webs.iiitd.edu.in/raghava/toxinpred/pepsearch_S.php?seq=CGLPGTKCCK&thval=0.0) | Toxin |
| [TCLKSGAICH](https://webs.iiitd.edu.in/raghava/toxinpred/pepsearch_S.php?seq=TCLKSGAICH&thval=0.0) | Non-Toxin | [KSGAICHPVF](https://webs.iiitd.edu.in/raghava/toxinpred/pepsearch_S.php?seq=KSGAICHPVF&thval=0.0) | Non-Toxin | [GLPGTKCCKK](https://webs.iiitd.edu.in/raghava/toxinpred/pepsearch_S.php?seq=GLPGTKCCKK&thval=0.0) | Toxin |
| [CLKSGAICHP](https://webs.iiitd.edu.in/raghava/toxinpred/pepsearch_S.php?seq=CLKSGAICHP&thval=0.0) | Toxin | [SGAICHPVFC](https://webs.iiitd.edu.in/raghava/toxinpred/pepsearch_S.php?seq=SGAICHPVFC&thval=0.0) | Non-Toxin | [LPGTKCCKKP](https://webs.iiitd.edu.in/raghava/toxinpred/pepsearch_S.php?seq=LPGTKCCKKP&thval=0.0) | Toxin |
| [LKSGAICHPV](https://webs.iiitd.edu.in/raghava/toxinpred/pepsearch_S.php?seq=LKSGAICHPV&thval=0.0) | Non-Toxin | [GAICHPVFCP](https://webs.iiitd.edu.in/raghava/toxinpred/pepsearch_S.php?seq=GAICHPVFCP&thval=0.0) | Toxin | [AICHPVFCPR](https://webs.iiitd.edu.in/raghava/toxinpred/pepsearch_S.php?seq=AICHPVFCPR&thval=0.0) | Non-Toxin |
